# Clinical impact of an integrated e-health system for diabetes self-management support and shared decision making (POWER2DM): a randomised controlled trial

**DOI:** 10.1007/s00125-023-06006-2

**Published:** 2023-09-29

**Authors:** Merel M. Ruissen, José D. Torres-Peña, Bas S. Uitbeijerse, Antonio P. Arenas de Larriva, Sasja D. Huisman, Tuncay Namli, Eckhard Salzsieder, Lutz Vogt, Manuela Ploessnig, Bob van der Putte, Armelle Merle, Gustavo Serra, Gustavo Rodríguez, Albert A. de Graaf, Eelco J. P. de Koning, Javier Delgado-Lista, Jacob K. Sont, POWER2DM Consortium

**Affiliations:** 1 Department of Medicine, Leiden University Medical Center, Leiden, the Netherlands; 2 Department of Biomedical Data Sciences, Medical Decision Making Section, Leiden University Medical Center, Leiden, the Netherlands; 3 Lipids and Atherosclerosis Unit, Department of Internal Medicine, Reina Sofía University Hospital, Córdoba, Spain; 4 Department of Medical and Surgical Sciences, University of Córdoba, Córdoba, Spain; 5 Maimonides Biomedical Research Institute of Córdoba, Córdoba, Spain; 6 Centro de Investigación Biomédica en Red Fisiopatologia de la Obesidad y Nutricion (CIBEROBN), Instituto de Salud Carlos III, Madrid, Spain; 7 SRDC Software Research & Development and Consultancy Corp., Ankara, Turkey; 8 Institute of Diabetes ‘Gerhardt Katsch’, Karlsburg, Germany; 9 Diabetes Service Center GmbH, Karlsburg, Germany; 10 Salzburg Research Forschungsgesellschaft, Salzburg, Austria; 11 Primedata, Delft, the Netherlands; 12 iHealthLabs Europe, Paris, France; 13 Netherlands Organization for Applied Scientific Research (TNO), Utrecht, the Netherlands

**Keywords:** Diabetes, e-health, Glycaemic control, m-health, Quality of life, RCT, Self-management, Shared decision making

## Abstract

**Aims/hypothesis:**

There is a lack of e-health systems that integrate the complex variety of aspects relevant for diabetes self-management. We developed and field-tested an e-health system (POWER2DM) that integrates medical, psychological and behavioural aspects and connected wearables to support patients and healthcare professionals in shared decision making and diabetes self-management.

**Methods:**

Participants with type 1 or type 2 diabetes (aged >18 years) from hospital outpatient diabetes clinics in the Netherlands and Spain were randomised using randomisation software to POWER2DM or usual care for 37 weeks. This RCT assessed the change in HbA_1c_ between the POWER2DM and usual care groups at the end of the study (37 weeks) as a primary outcome measure. Participants and clinicians were not blinded to the intervention. Changes in quality of life (QoL) (WHO-5 Well-Being Index [WHO-5]), diabetes self-management (Diabetes Self-Management Questionnaire – Revised [DSMQ-R]), glycaemic profiles from continuous glucose monitoring devices, awareness of hypoglycaemia (Clarke hypoglycaemia unawareness instrument), incidence of hypoglycaemic episodes and technology acceptance were secondary outcome measures. Additionally, sub-analyses were performed for participants with type 1 and type 2 diabetes separately.

**Results:**

A total of 226 participants participated in the trial (108 with type 1 diabetes; 118 with type 2 diabetes). In the POWER2DM group (*n*=111), HbA_1c_ decreased from 60.6±14.7 mmol/mol (7.7±1.3%) to 56.7±12.1 mmol/mol (7.3±1.1%) (means ± SD, *p*<0.001), compared with no change in the usual care group (*n*=115) (baseline: 61.7±13.7 mmol/mol, 7.8±1.3%; end of study: 61.0±12.4 mmol/mol, 7.7±1.1%; *p*=0.19) (between-group difference 0.24%, *p*=0.008). In the sub-analyses in the POWER2DM group, HbA_1c_ in participants with type 2 diabetes decreased from 62.3±17.3 mmol/mol (7.9±1.6%) to 54.3±11.1 mmol/mol (7.1±1.0%) (*p*<0.001) compared with no change in HbA_1c_ in participants with type 1 diabetes (baseline: 58.8±11.2 mmol/mol [7.5±1.0%]; end of study: 59.2±12.7 mmol/mol [7.6±1.2%]; *p*=0.84). There was an increase in the time during which interstitial glucose levels were between 3.0 and 3.9 mmol/l in the POWER2DM group, but no increase in clinically relevant hypoglycaemia (interstitial glucose level below 3.0 mmol/l). QoL improved in participants with type 1 diabetes in the POWER2DM group compared with the usual care group (baseline: 15.7±3.8; end of study: 16.3±3.5; *p*=0.047 for between-group difference). Diabetes self-management improved in both participants with type 1 diabetes (from 7.3±1.2 to 7.7±1.2; *p*=0.002) and those with type 2 diabetes (from 6.5±1.3 to 6.7±1.3; *p*=0.003) within the POWER2DM group. The POWER2DM integrated e-health support was well accepted in daily life and no important adverse (or unexpected) effects or side effects were observed.

**Conclusions/interpretation:**

POWER2DM improves HbA_1c_ levels compared with usual care in those with type 2 diabetes, improves QoL in those with type 1 diabetes, improves diabetes self-management in those with type 1 and type 2 diabetes, and is well accepted in daily life.

**Trial registration:**

ClinicalTrials.gov NCT03588104.

**Funding:**

This study was funded by the European Union’s Horizon 2020 Research and Innovation Programme (grant agreement number 689444).

**Graphical Abstract:**

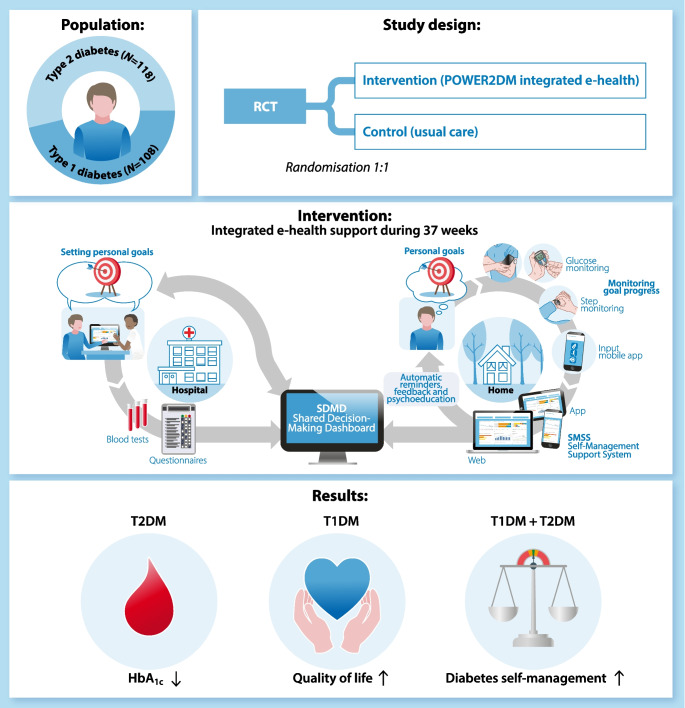

**Supplementary Information:**

The online version contains supplementary material available at 10.1007/s00125-023-06006-2.


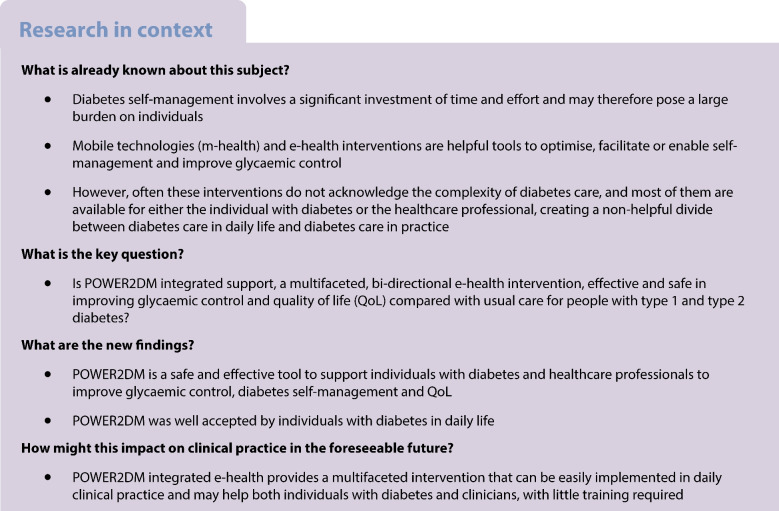


## Introduction

Diabetes mellitus imposes a major disease burden on both individuals and healthcare systems [1]. The goals of treatment for diabetes are to prevent or delay complications and optimise quality of life (QoL) [2]. To prevent diabetes-related complications, blood glucose values need to be kept as close to normal as possible using medication, diet, physical activity and glucose monitoring [3–5]. Treatment and self-management plans should be created in consultation with people with diabetes based on their individual preferences, values and goals [2]. Diabetes self-management involves a significant investment of time and effort, and may therefore pose a large burden on individuals, both practically and emotionally [6, 7]. Consequently, psychological issues related to diabetes outcomes and barriers to diabetes self-management are commonly observed [8, 9], resulting in suboptimal self-management, a reduction in QoL or poor healthcare outcomes [7]. Despite self-management support now being acknowledged as one of the most important factors in diabetes care [10], healthcare systems often still focus on biomedical outcomes and screening for complications, rather than on the burden of disease and potential barriers to self-management, or facilitating support and strategies that help improve patient empowerment [11, 12]. This results in a divide between patients’ needs and the healthcare support provided [13, 14].

Acknowledging patients’ needs for more self-management support, a variety of mobile technologies (m-health) and e-health interventions have been developed [15–19] that have often been shown to be accepted by patients as a helpful tool to optimise, facilitate or enable self-management and improve glycaemic control [20–22]. However, most of these interventions involve ‘stand-alone’ systems or apps that are used by patients but are not accessible to healthcare professionals. These fragmented applications, which often only focus on one specific aspect such as carbohydrate intake, exercise or glucose monitoring, do not acknowledge the complexity of self-management and impede the uptake of such systems and use of the resulting data in standard diabetes care. Therefore there is a need for integrated digital systems that support all aspects of diabetes (self-)management, facilitate shared decision making (SDM) between patients and healthcare professionals, and enable integration of behavioural, psychological and medical data in diabetes care.

To fulfil this need and provide both patients and healthcare professionals with a digital tool to facilitate self-management (support) and SDM, we developed the POWER2DM integrated e-health support system. This self-management support system collects, integrates and presents a variety of data in a dashboard for patients and healthcare professionals, supports patients in self-management in daily life, and creates insights into potential barriers, behaviours and outcomes. This information may help patients and healthcare professionals to collaborate and engage in SDM. As people with type 1 and type 2 diabetes have different needs and require different types of support, the POWER2DM support system aims to be flexible, patient-centred and adjustable by individuals themselves to their wishes and needs.

The aim of this study was to assess whether the POWER2DM integrated e-health support system is effective and safe in improving glycaemic control and QoL compared with usual care for people with type 1 or type 2 diabetes.

## Methods

### Overall design

This RCT (NCT03588104, ClinicalTrials.gov) aimed to test the effectiveness and safety of an integrated e-health system (POWER2DM) to support individuals with diabetes and healthcare professionals in diabetes self-management and SDM compared with usual care during 37 weeks of follow-up. The study was performed using the same methods in both the Netherlands and Spain. The study was approved by the Medical Ethical Committee Leiden/Den Haag/Delft under the Medical Research Involving Human Subjects Act, and by the Research Ethics Committee of Reina Sofía University Hospital as part of the Sistema Sanitario Público de Andalucía Research Ethics Committee Network, and complies with the Declaration of Helsinki.

### The POWER2DM integrated e-health system

**Fig. 1:**
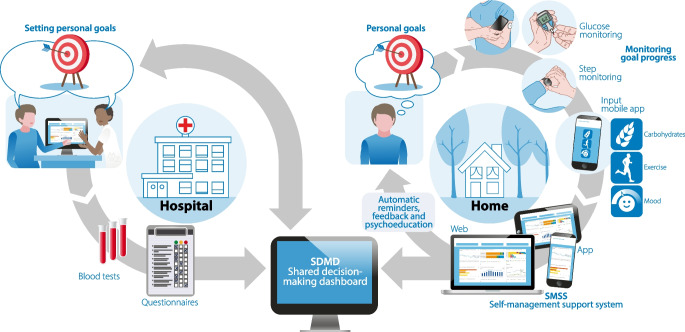
POWER2DM intervention. Personal goal setting is first carried out by the participant and the healthcare professional using SDM and the data from the POWER2DM SDMD during clinical consultation. The participant then tries to reach these personal goals through use of the POWER2DM SMSS and multiple connected wearables, while receiving automatic reminders, feedback and psychoeducation

The POWER2DM integrated e-health system is a clinical-based support system that was developed to create insight into an individual’s medical, behavioural and psychological data, to support the individual and healthcare professionals collaborating in SDM and creating a treatment plan that fits the individual’s situation, and to support the individual in daily life to reach their self-management goals (Fig. 1). The system has two components: the web-based shared decision-making dashboard (SDMD) (ESM Fig. 1), used by individuals together with healthcare professionals during clinical consultations, and a self-management support system (SMSS) [23] that is available as a mobile application (ESM Fig. 2) and webpage (ESM Fig. 3) for people to use at home and in daily life. Clinical consultations were performed by diabetes nurses and clinicians who were part of the study team, and who were self-trained (using an instruction guide and by trial and error) to work with the technological systems involved. Individuals were instructed on how to use the SMSS by the nurse/clinician who performed the randomisation visit, and online support videos were available to use at home.

#### The POWER2DM SDMD

The SDMD is a tool for healthcare professionals and individuals to use together during clinical consultations. It provides a visual overview of medical, behavioural and psychological data gathered by the individual. These data may be manually entered into the mobile app, such as blood glucose values, carbohydrate intake or exercise, or collected by connected wearables that were provided to participants as part of the intervention. Physical activity was measured using a Fitbit Charge 2 (Fitbit Health Solutions, USA), blood glucose values were measured using an iHealth BG5 glucometer (iHealthlabs, Australia), and interstitial glucose values were measured using blinded (Freestyle Libre Pro) or unblinded (Freestyle Libre) continuous glucose monitoring devices (Abbott Laboratories, USA). The structured visual data overview in the SDMD aims to help individuals and healthcare professionals to obtain valuable insights about the individuals’ situation and reveal potential targets for improvement. Furthermore, the SDMD automatically identifies potential barriers to self-management based on behavioural data entered in the mobile app and the outcomes of questionnaires that the participants filled in during study visits.

#### Web-based and mobile POWER2DM SMSS

The SMSS consists of a webpage and mobile app for individuals to use during their daily life to set goals, track their goal progress, and receive support to reach their goals. Goals are set by individuals and healthcare professionals together during clinical consultations using the SDMD, or by the individual alone using the SMSS webpage. The SDMD and SMSS automatically transfer the goals to the mobile app. The mobile app then combines manually entered data and data from the connected wearables that were provided to participants as part of the intervention to automatically track goal progress over time, and send reminders for planned tasks (ESM Methods 1). If the SMSS registers that an individual has failed to complete a pre-planned task, it automatically refers them to the SMSS webpage and guides them through a barrier identification process to identify potential issues preventing them from reaching their goal(s). If barriers for self-management are detected, targeted interventions, psychological exercises and psychoeducation are automatically provided by the webpage to help overcome these barriers. Alternatively, individuals can choose to adapt their self-management goals.

### The POWER2DM intervention

The POWER2DM intervention comprised a non-protocolised, multifaceted intervention, combining the use of the POWER2DM integrated e-health system with SDM and personal goal setting during clinical consultations, and manual and automated data collection, overview and feedback (Fig. 1). Participants were allowed to use the elements of the support system as they saw fit, in line with their self-management goals.

#### Population

People with type 1 or type 2 diabetes who were receiving care at the hospital outpatient diabetes clinics of the Leiden University Medical Center and affiliated teaching hospitals or the Reina Sofía University Hospital were eligible for participation if they fulfilled the following inclusion criteria: age ≥18 years, ability to self-monitor and work with a computer and smartphone with internet connection, sufficient language comprehension and the ability to complete questionnaires. People who were eligible for participation were proactively identified at the outpatient clinic and asked to participate. A more detailed description of inclusion and exclusion criteria is given in ESM Methods 2.

#### Randomisation, interventions, subsequent care and follow-up visits

This RCT consisted of a data collection and handling period of 4 weeks, and three consecutive intervention periods of 11 weeks (total duration 37 weeks) (ESM Fig. 4). After providing informed consent, participants were randomised in a 1:1 ratio to either the POWER2DM group or the usual care group in strata of equal size for type 1 or type 2 diabetes using randomisation software (Castor EDC, Castor, the Netherlands). The primary outcome was the difference in change in HbA_1c_ between the POWER2DM and usual care groups during the study period. Secondary outcomes analysed in this paper were changes in QoL (assessed using the WHO-5 Well-Being Index [WHO-5] [24]), diabetes self-management (assessed using the Diabetes Self-Management Questionnaire – Revised [DSMQ-R] [25]), glycaemic profiles obtained using continuous glucose monitoring devices, hypoglycaemia awareness (assessed using the Clarke hypoglycaemia unawareness instrument [26]), number of hypoglycaemic episodes and technology acceptance (assessed using the Technology Acceptance Questionnaire [27]; see ESM Technology Acceptance Questionnaire [TAQ]). A more detailed description of the outcomes measured and a complete list of secondary outcome measures are given in ESM Methods 3.

To assess glycaemic control, each participant in the POWER2DM and usual care groups was provided with a blinded continuous glucose monitor for 2 consecutive weeks at the start of the study (weeks 0–2) and the end of the study (weeks 35–37). The study visits for participants included in the POWER2DM group focused on SDM and goal setting for self-management behaviour, using the POWER2DM integrated e-health system. Clinical information about glycaemic control and diabetes-related outcomes was gathered, and laboratory tests, anthropometric measurements and questionnaires were completed at baseline (week 0), week 11, week 22 and week 37. At week 4, week 15 and week 26, all gathered information was used by the clinicians and participants to engage in SDM and set personalised treatment goals together. The participants would then try to achieve these goals with the help of the mobile application and webpage of the SMSS, which they used whenever they felt appropriate. Twice during the study (weeks 11–13 and 22–24), participants in the POWER2DM group received a non-blinded intermittently scanned continuous glucose monitoring device (FreeStyle Libre) to provide an additional learning opportunity and mimic real-life clinical practice, in which measurements from intermittently scanned continuous glucose monitoring devices are widely available and used. For participants in the usual care group, regular care visits with their usual diabetes care team were continued, together with reporting on glycaemic control and diabetes-related outcomes, laboratory tests, anthropometric measurements and questionnaires at baseline (week 0), week 11, week 22 and week 37. ESM Fig. 4 gives details of the visits in each group.

### Statistical methods

Details regarding sample size and power calculations are given in ESM Methods 4. Analyses were performed from an intention-to-treat perspective. Missing data were handled by multiple imputation (five imputed datasets) by chained equations. Stata version 16 (StataCorp, USA) was used to perform all analyses. All outcomes from the participant and clinical perspective were analysed using the Stata mixed command for multi-level linear regression. For all outcomes, we performed an overall analysis of all participants (participants with type 1 and type 2 diabetes combined) as well as subsequent separate analyses for participants with type 1 or type 2 diabetes. Data in the text are reported as means ± SD. A *p* value <0.05 was considered statistically significant. A more detailed description of the statistical analyses performed is given in ESM Methods 4.

## Results

**Fig. 2:**
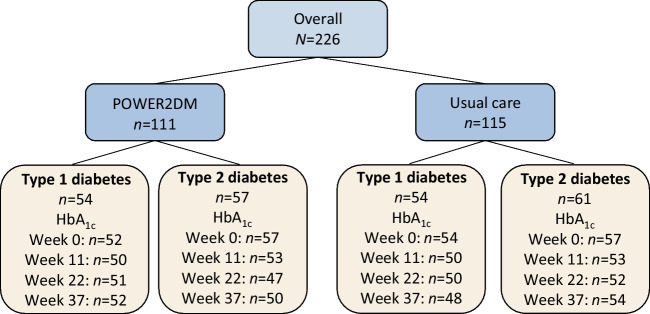
Flow chart showing the number of study participants in each group and the number for whom HbA_1c_ data were available at each time point

A total of 226 participants with diabetes were recruited from outpatient clinics in the Netherlands and Spain, including 108 from Leiden University Medical Center and affiliating teaching hospitals (83 with type 1 diabetes; 25 with type 2 diabetes) and 118 from Reina Sofía University Hospital, Córdoba, Spain (25 with type 1 diabetes; 93 with type 2 diabetes). Of these, 111 were randomised to the POWER2DM group and 115 to the usual care group (Table 1 and Fig. 2). Participants had a mean age of 51.3±12.0 years, and 36.3% were female. In total, 25.2% were already monitoring their glucose values using (intermittently scanned) continuous glucose monitoring devices prior to the start of the study. The mean follow-up duration was 40.2±4.7 weeks. Baseline characteristics were similar in the POWER2DM and usual care groups (Table 1).

**Table 1:** Baseline characteristics

Characteristic	Total group		Type 1 diabetes		Type 2 diabetes	
	POWER2DM	Usual care	POWER2DM	Usual care	POWER2DM	Usual care
*N*	111	115	54	54	57	61
Age, years	51.5±13.2	51.1±10.9	44.6±13.9	45.3±11.5	57.8±8.6	56.2±7.2
Female	40 (36)	42 (37)	19 (35)	25 (46)	21 (37)	17 (28)
BMI, kg/m^2^	29.1±5.9	28.8±5.0	26.3±5.2	26.0±3.8	31.8±5.3	31.2±4.7
Level of education						
Primary	21 (19)	21 (18)	1 (2)	2 (4)	20 (35)	19 (31)
Secondary/vocational	23 (21)	22 (19)	13 (24)	15 (28)	10 (18)	7 (11)
University	55 (50)	59 (51)	35 (65)	36 (67)	20 (35)	23 (38)
Unknown	12 (11)	13 (11)	5 (9)	1 (2)	7 (12)	12 (20)
Smoking	16 (14)	15 (13)	5 (9)	4 (7)	11 (19)	11 (18)
Duration of diabetes, years	16.9±11.6	17.9±12.3	21.7±12.7	23.3±13.5	11.9±7.6	12.1±7.6
Glucose-lowering medication						
Insulin	82 (74)	84 (73)	54 (100)	54 (100)	28 (49)	30 (49)
Metformin	40 (36)	60 (52)	2 (4)	6 (11)	38 (67)	54 (89)
GLP-1 receptor antagonist	3 (3)	4 (3)	0 (0)	0 (0)	3 (5)	4 (7)
SGLT-2 inhibitor	13 (12)	20 (17)	0 (0)	0 (0)	13 (23)	20 (33)
DPP-4 inhibitor	18 (16)	14 (12)	0 (0)	0 (0)	18 (32)	14 (23)
Sulfonylurea derivative	6 (5)	6 (5)	0 (0)	0 (0)	6 (11)	6 (10)
Pioglitazone	2 (2)	1 (1)	0 (0)	0 (0)	2 (4)	1 (2)
Other	3 (3)	3 (3)	0 (0)	0 (0)	3 (5)	3 (5)
Glucose monitoring						
None	27 (24)	27 (23)	0 (0)	0 (0)	27 (47)	27 (44)
Yes	84 (76)	88 (77)	54 (100)	54 (100)	30 (53)	34 (56)
Blood glucose monitoring only	55	60	33	26	22	34
Continuous glucose monitoring	8	3	2	3	6	0
Intermittently scanned continuous glucose monitoring	21	25	19	25	2	0
Complications						
None	56 (50)	63 (55)	23 (43)	21 (39)	33 (58)	42 (69)
Retinopathy	42 (38)	43 (37)	29 (54)	31 (57)	13 (23)	12 (20)
Laser coagulation	8 (7)	8 (7)	4 (7)	6 (11)	4 (7)	2 (3)
Diabetic neuropathy	12 (11)	16 (14)	7 (13)	9 (17)	5 (9)	7 (11)
Diabetic nephropathy	7 (6)	7 (6)	5 (9)	2 (4)	2 (4)	2 (3)
Macroangiopathy (peripheral vascular disorders)	19 (17)	11 (10)	7 (13)	3 (6)	12 (21)	8 (13)
Comorbidity	65 (59)	79 (69)	36 (67)	46 (85)	29 (51)	33 (54)
BP, mmHg						
Systolic	133±18	133±25	131±16	128±20	136±20	137±28
Diastolic	79±10	78±9	80±9	77±10	79±11	79±8
Lipids, mmol/l						
Total cholesterol	4.45±0.92	4.65±0.94	4.42±0.81	4.89±0.73	4.45±1.03	4.45±1.06
LDL-cholesterol	2.41±0.77	2.59±0.85	2.37±0.62	2.74±0.71	2.45±0.88	2.46±0.94
HDL-cholesterol	1.41±0.44	1.45±0.47	1.66±0.44	1.73±0.45	1.17±0.28	1.20±0.31
Triglycerides	1.50±2.13	1.39±1.13	0.83±0.36	0.94±0.60	2.13±2.82	1.78±1.32

Values are means ± SD or *n* (%)

Information about sex (female/male) was self-reported by participants

DPP-4, dipeptidylpeptidase-4; GLP-1, glucagon-like peptide-1; SGLT-2, sodium–glucose cotransporter-2

Overall, of the 226 participants included in the study, 108 had type 1 diabetes and 118 had type 2 diabetes (Table 1). Individuals with type 2 diabetes had a higher BMI (31.5±5.0 kg/m^2^) than those with type 1 diabetes (26.1±4.6 kg/m^2^). Individuals with type 1 diabetes had more diabetes-related complications than those with type 2 diabetes (59.3% and 36.4%, respectively). Of the participants with type 1 diabetes, 45.8% monitored their glucose values using a (intermittently scanned) continuous glucose monitoring device, compared with 12.5% of those with type 2 diabetes.

### Glycaemic control

**Fig. 3:**
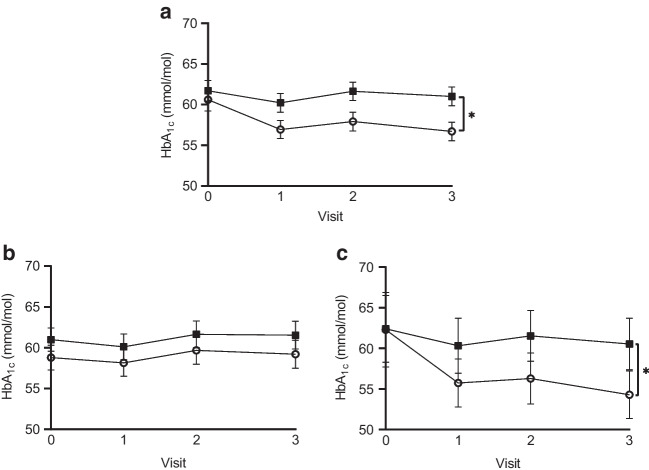
HbA_1c_ values at baseline and during follow-up. (**a**) HbA_1c_ values for the entire group (POWER2DM: *n*=111; usual care: *n*=115). (**b**) HbA_1c_ values for participants with type 1 diabetes (POWER2DM: *n*=54; usual care: *n*=54). (**c**) HbA_1c_ values for participants with type 2 diabetes (POWER2DM: *n*=57; usual care: *n*=61). Data are means and 95% CI. Open circles represent the POWER2DM group; black squares represent the usual care group. *p <0.05 between groups

In the POWER2DM group, HbA_1c_ decreased from 60.6±14.7 mmol/mol (7.7±1.3%) to 56.7±12.1 mmol/mol (7.3±1.1%) during the study (*p*<0.001). No significant change in HbA_1c_ was observed in the usual care group (baseline: 61.7±13.7 mmol/mol, 7.8±1.3%; end of study: 61.0±12.4 mmol/mol, 7.7±1.1%; *p*=0.19) (Fig. 3a). The improvement in HbA_1c_ in the POWER2DM group was already present at 3 months, was maintained over time and was 2.6 mmol/mol (0.24%) greater than in the usual care group (between-group difference: *p*=0.008). Within the POWER2DM group, the HbA_1c_ level of participants with type 2 diabetes improved over the course of the study (baseline: 62.3±17.3 mmol/mol, 7.9±1.6%; end of study: 54.3±11.1 mmol/mol, 7.1±1.0%; *p*<0.001) (between-group difference: −5.2 mmol/mol (0.48%), *p*=0.01) (Fig. 3c), compared with no change in HbA_1c_ level in those with type 1 diabetes in the POWER2DM group (baseline: 58.8 ± 11.2 mmol/mol, 7.5±1.0%; end of study: 59.2±12.7 mmol/mol, 7.6±1.2%; *p*=0.84) (between-group difference: 0.1 mmol/mol (0.01%), *p*=0.88) (Fig. 3b).

Glucose profiles obtained from blinded continuous glucose monitors showed no significant change in time in range (3.9–10.0 mmol/l) for the POWER2DM group (baseline: 62.8±20.5%; end of study: 68.2±19.7%; *p*=0.053); however, a significant improvement in time between 10.0 and 13.9 mmol/l was observed (baseline: 21.6±11.6%; end of study: 17.9±12.1%; *p*=0.001), together with a small but significant increase in time between 3.0 and 3.9 mmol/l (baseline: 3.7±3.8%; end of study: 6.3±6.0%, *p*<0.001). The percentage of time above 13.9 mmol/l and below 3.0 mmol/l did not change significantly in the POWER2DM group during the trial. The usual care group showed a similar effect, with an increase in time in range (3.9–10.0 mmol/l) (baseline: 59.3±22.4%; end of study: 64.5±21.2%; *p*=0.024), a decrease in both time between 10.0 and 13.9 mmol/l (baseline: 22.0±11.7%; end of study: 17.3 ± 12.3%; *p*=0.007) and time above 13.9 mmol/l (baseline: 14.7±17.6%; end of study: 7.4±11.0%; *p*<0.001), and an increase in time between 3.0 and 3.9 mmol/l (baseline: 3.9±3.9%; end of study: 5.6±5.3%; *p*=0.003) and time below 3.0 mmol/l (baseline: 2.8±4.2%; end of study: 4.5±6.5%; *p*=0.004) (Table 2).

**Table 2:** Outcomes of blinded continuous glucose monitoring

Variable	Overall				Type 1 diabetes				Type 2 diabetes			
	POWER2DM		Usual care		POWER2DM		Usual care		POWER2DM		Usual care	
	Baseline	End	Baseline	End	Baseline	End	Baseline	End	Baseline	End	Baseline	End
Time below 3.0 mmol/l	2.7±5.2	3.4±3.9	2.8±4.2	4.5±6.5*	4.7±6.7	4.0±3.9	4.5±5.2	4.9±5.9	0.9±1.7	2.7±3.9	1.3±2.2	4.1±6.9*
Time in range 3.0–3.9 mmol/l	3.7±3.8	6.3±6.0*	3.9±3.9	5.6±5.3*	5.4±4.3	5.8±4.0	5.0±3.9	5.4±4.2	2.1±2.4	6.7±7.5*	2.9±3.7	5.8±6.2*
Time in range 3.9–10.0 mmol/l	62.8±20.5	68.2±19.7	59.3±22.4	64.5±21.2*	55.6±15.6	56.6±14.2	52.6±18.9	56.8±16.4*	69.6±22.2	79.2±17.9*	65.2±23.8	71.3±22.7
Time in range 10.0–13.9 mmol/l	21.6±11.6	17.9±12.1*	22.0±11.7	17.3±12.3*	23.3±9.1	22.8±9.4	23.3±10.1	19.6±10.9*	19.9±13.4	13.3±12.6*	20.8±12.8	15.3±13.3*
Time above 13.9 mmol/l	11.4±12.9	9.1±11.6†	14.7±17.6	7.4±11.0*	15.3±13.5	12.9±11.2†	18.0±16.8	9.0±11.2*	7.8±11.2	5.6±11.0	11.8±17.8	6.1±10.7*
Mean no. of days recorded	12.4	14.0	12.3	12.7	11.8	14.0	12.1	13.9	13.0	14.0	12.5	11.6

Outcomes of blinded continuous glucose monitoring during 2 weeks in participants with type 1 or type 2 diabetes receiving POWER2DM or usual care at baseline and at the end of the study. Data represent the time below range, time in range and time above range as a percentage of the total time that the glucose monitoring device was worn by participants (means ± SD)

^*^p<0.05 for change in percentage of time within each range between baseline and the end of the study within the POWER2DM or usual care groups

^†^*p*<0.05 for change in percentage of time within each range between the POWER2DM group and the usual care group

In participants with type 1 diabetes, the improvements in time in range and time above range and also the slight increase in time below range were less pronounced than the differences in glucose profiles over time found in participants with type 2 diabetes (Table 2 and ESM Fig. 5).

### BMI

Overall, BMI did not change over time in the POWER2DM group (baseline: 29.3±5.8 kg/m^2^; end of study: 29.2±5.7 kg/m^2^; *p*=0.13) or in the usual care group (baseline: 28.8±4.8 kg/m^2^; end of study: 28.8±4.6 kg/m^2^; *p*=0.54) (between-group difference: *p*=0.13). Additionally, no change in BMI was observed over time in participants with type 1 diabetes in the POWER2DM group (baseline: 26.4±5.2 kg/m^2^; end of study: 26.5±5.2 kg/m^2^; *p*=0.98) or the usual care group (baseline: 25.8±3.5 kg/m^2^; end of study: 26.2±3.5 kg/m^2^; *p*=0.10) (between-group difference: *p*=0.27), or in those with type 2 diabetes in the POWER2DM group (baseline: 32.1±5.0 kg/m^2^; end of study: 31.8±4.8 kg/m^2^; *p*=0.09) or the usual care group (baseline: 31.4±4.3 kg/m^2^; end of study: 31.2±4.2 kg/m^2^; *p*=0.74) (between-group difference: *p*=0.28).

### Lipids

The changes in total cholesterol, LDL-cholesterol, HDL-cholesterol and triglycerides did not differ between the POWER2DM and usual care groups (*p*>0.51), nor when analysed separately for participants with type 1 diabetes (*p*>0.15) and those with type 2 diabetes (*p*>0.18) (ESM Table 1).

### Safety

Overall, the time spent between 3.0 and 3.9 mmol/l increased in the POWER2DM group (from 3.7±3.8% to 6.3±6.0%; *p*<0.001) without a significant increase in time below 3.0 mmol/l (from 2.7±5.2% to 3.4±3.9%; *p*=0.43). The increase in time between 3.0 and 3.9 mmol/l was not associated with clinical symptoms reported by participants or with severe hypoglycaemic episodes, nor was it associated with an increase in impaired awareness of hypoglycaemia, as measured by the Clarke hypoglycaemia unawareness instrument (overall: −0.07, *p*=0.23; type 1 diabetes: −0.08, *p*=0.36; type 2 diabetes: 0.02, *p*=0.76).

### QoL and self-management

**Fig. 4:**
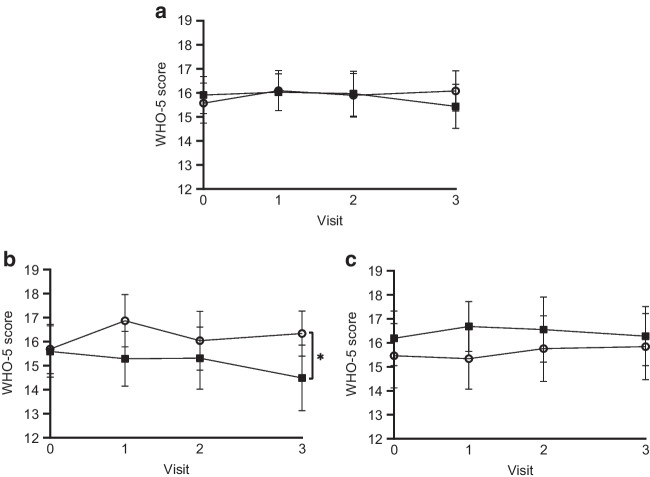
WHO-5 scores (possible range 0–25) for QoL over the course of the study. (**a**) WHO-5 scores for the entire group (POWER2DM: *n*=111; usual care: *n*=115). (**b**) WHO-5 scores for participants with type 1 diabetes (POWER2DM: *n*=54; usual care: *n*=54). (**c**) WHO-5 scores for participants with type 2 diabetes (POWER2DM: *n*=57; usual care: *n*=61). Data are means and 95% CI. Open circles represent the POWER2DM group; black squares represent the usual care group. *p<0.05 between groups

Overall scores for QoL (WHO-5) did not change in either the POWER2DM or the usual care group (Fig. 4a). However, in participants with type 1 diabetes, there was an improvement in QoL in the POWER2DM group compared with the usual care group (between-group difference: *p*=0.047) (Fig. 4b).

Overall diabetes self-management scores, reflected by the DSMQ-R questionnaire, improved both in the POWER2DM group (from 6.9±1.3 to 7.2±1.3; *p*<0.001) and in the usual care group (from 6.7±1.5 to 7.0±1.4); *p*=0.006) (between-group difference: *p*=0.21) (ESM Fig. 6). In participants with type 1 diabetes, an improvement in DSMQ-R scores over time was found both in the POWER2DM group (from 7.3±1.2 to 7.7±1.2; *p*=0.002) and in the usual care group (from 7.0±1.5 to 7.4±1.4; *p*=0.009). There was no significant difference between the groups (between-group difference: *p*=0.55) (ESM Fig. 6b). In participants with type 2 diabetes, there was an improvement in DSMQ-R scores in the POWER2DM group (from 6.5±1.3 to 6.7±1.3; *p*=0.003) but not in the usual care group (from 6.4±1.4 to 6.6±1.2; *p*=0.15). There was no significant difference between these groups (*p*=0.33) (ESM Fig. 6c). Scores for self-monitoring of blood glucose values improved in participants with type 1 diabetes and those with type 2 diabetes in the POWER2DM group, but not in the usual care group (ESM Fig. 7a); however, only in participants with type 2 diabetes was there a significant difference between the POWER2DM and usual care groups (between-group difference: *p*=0.036) (ESM Fig. 7c).

### Use of the POWER2DM system

System usage was highest in period 1 (weeks 4–15: 1.05 times per day) and gradually decreased with time towards the end of the study period (period 3, weeks 26–37: 0.41 times per day; *p*=0.001). Overall, system usage by participants with type 2 diabetes was significantly lower than that by participants with type 1 diabetes (between-group difference: −0.54 times per day; *p*<0.001). Participant satisfaction, as assessed using the Technology Acceptance Questionnaire, was high in both those with type 1 diabetes and those with type 2 diabetes, with positive scores in ten of the ten domains, indicating that the system was well accepted by participants in their daily diabetes care (ESM Fig. 8).

## Discussion

This RCT shows that POWER2DM integrated e-health support improved glycaemic control, QoL and self-management in people with diabetes mellitus, without increasing clinically relevant hypoglycaemia (blood glucose <3.0 mmol/l). POWER2DM integrated e-health support was well accepted in daily life by both those with type 1 diabetes and those with type 2 diabetes.

Within the POWER2DM group, outcomes of blinded continuous glucose monitoring showed a decrease in time above range, together with a slight increase in time between 3.0 and 3.9 mmol/l, but no increase in clinically relevant hypoglycaemia (time below 3.0 mmol/l). As baseline glycaemic control was good in the POWER2DM group, with a mean HbA_1c_ level of 60.6±14.7 mmol/mol (7.7 ±1.3%), the slight increase in time spent between 3.0 and 3.9 mmol/l may be expected. In the usual care group, a decrease in time above range, an increase in time within range and an increase in time below range were found, but no change in HbA_1c_. An explanation for this may be that use of the blinded continuous glucose monitor for 2 weeks resulted in a short-lived emphasis on glycaemic control that was not reflected in changes in HbA_1c_.

The sub-analyses in our study indicated that the improvement in HbA_1c_, associated with improvements in glucose monitoring outcomes, was more pronounced in those with type 2 diabetes, and was already established within the first 3 months, after which the beneficial effect was sustained. As education has been shown to be directly associated with diabetes knowledge [28] and participants with type 2 diabetes in our study had received a lower level of prior education regarding their diabetes than those with type 1 diabetes, it is likely that those with type 2 diabetes experienced a steeper learning curve. A study by Feigerlová et al also found no effect of additional e-health education on HbA_1c_ levels in people with type 1 diabetes [29], supporting this hypothesis.

Previous studies on the effects of m-health and e-health interventions have reported similar findings of improved glycaemic control in people with type 1 diabetes [30] and type 2 diabetes [31, 32], decreased feelings of distress [30, 33] and improved QoL [33, 34]. A systematic review by Pal et al found no effect of m-health interventions on behavioural, emotional or cognitive outcomes [35]. However, the m-health interventions used were one-sided and were not combined with real-life clinical visits. Greenwood et al showed that the most effective strategy to support individuals is to use a two-way communication system, providing tailored support and individualised feedback [31]. Despite this evidence, m-health and e-health interventions are often one-sided, and frequently available to either the individual with diabetes (most often) or the healthcare professional, not incorporating real-life human interaction and creating a divide between diabetes care in practice and at home. This divide is not helpful when aiming for person-centred care, which requires collaboration between the individual with diabetes, as the expert on their life and living, and the clinician, as a medical expert. A helpful collaboration can only be established based on a meaningful connection, something that requires human contact, emphasising the need to combine m-health and e-health interventions with human contact and face-to-face clinical consultations.

POWER2DM integrated e-health support distinguishes itself from other m-health and e-health systems by providing multifactorial support for both individuals with diabetes and healthcare professionals. However, the incorporation of multiple electronic interfaces, several connected devices and specific goal-oriented consultations with healthcare professionals makes it difficult to determine the effect of specific components of POWER2DM. Thus the effect of POWER2DM can only be evaluated as a whole, acknowledging that both an increase in consultation frequency [36] and the use of intermittently scanned continuous glucose monitoring devices [37, 38] improve glycaemic control and also decrease diabetes distress [38] and improve QoL [36]. While the additional effect of use of intermittently scanned continuous glucose monitoring devices [39] as a part of the POWER2DM intervention should be taken into account, HbA_1c_ levels had already improved before the use of these monitoring devices, and this device was only available twice for 2 weeks, limiting the expected effect. Furthermore, the use of activity trackers such as Fitbits has shown to result in an increase in physical activity and weight loss, which may also improve glycaemic control and psychological outcomes [40]. We believe the multifaceted character of the system to be one of the major strengths of this study, as it not only acknowledges the complexity of diabetes care, but also fits in with the current state-of-the-art multifactorial care approach. This care approach aims to address all factors that may affect healthcare outcomes and to support the day-to-day decision making, planning, monitoring, evaluation and problem-solving involved in diabetes self-management through a multistep model. Through the various functions, the system is able to gather information about and intervene in a broad variety of behavioural, psychological and medical aspects of an individual’s self-management that ultimately determine glycaemic control and QoL.

A limitation to this study is the fact that participants were not blinded to the intervention, so expectation bias cannot be ruled out. However, we observed the same effect size in objective outcomes such as HbA_1c_ level and in more subjective outcomes such as diabetes self-management and QoL, aspects of diabetes that have been shown to all be connected [41]. Another limitation is that the POWER2DM integrated e-health support system is less easily accessible for older people, people experiencing vision loss and people with limited technological skills or devices, and for clinical use in low-income countries or other clinical fields in which a computer is not always readily available. However, with the rapid technological advances, the group of older people who are capable of using this modern technology is growing, and the number of people owning a smartphone in low-income countries is increasing. With its adjustable character and person-centred clinical consultations focused on SDM and personal goal setting, the POWER2DM integrated e-health support system is expected to provide care that fits a broad range of people from a variety of backgrounds and socioeconomic situations, and with varying literacy and educational levels.

While implementation of the POWER2DM integrated e-health support system in standard care may initially require a financial investment in software and an investment of time spent teaching individuals how to use the system and interpret the results, we expect the system to be cost-effective in the long term. Studies have shown that educating people helps them understand the consequences of their self-management decisions and makes them feel empowered [42], thus motivating them and potentially improving therapy adherence. Furthermore, the system may help to identify and address potential barriers, which will help to overcome crucial problems hampering glycaemic control and improve QoL.

User engagement with the POWER2DM integrated e-health support system gradually declined over time, as is commonly observed for m-health systems [43]. Whether this is the result of a successful and lasting change in behaviour, for which support of the system is no longer needed, or a lack of user engagement remains unclear. To our knowledge, there are no studies available about the long-term implications of declining user engagement in e-health systems. Therefore, the long-term effects of the system should be investigated further, as well as its viability and applicability in different healthcare systems, different countries and different patient populations.

In conclusion, the POWER2DM integrated e-health support system is unique in its design, aiming to bridge the gap in diabetes care between the diabetes clinic and daily life. Its multifaceted approach acknowledges the complexity of the various domains of self-management and how these domains intertwine. It automatically identifies potential barriers to self-management, and provides practical tools and psychoeducation to overcome these barriers. This study showed that the POWER2DM system is a safe and effective tool to support patients and healthcare professionals to improve glycaemic control and self-management. The POWER2DM integrated e-health support system provides a multifaceted intervention that could be easily implemented into daily clinical practice and help both patients and clinicians, with little training required.

### Supplementary Information

Below is the link to the electronic supplementary material.

ESM (PDF 1.25 MB)

## Data Availability

Data are available from the corresponding author on reasonable request.

## Abbreviations

DSMQ-R: Diabetes Self-Management Questionnaire – Revised
QoL: Quality of life
SDM: Shared decision making
SDMD: Shared decision-making dashboard
SMSS: Self-management support system
WHO-5: WHO-5 Well-Being Index
