# Supplementary material for: Clinical impact of an integrated e-health system for diabetes self-management support and shared decision making (POWER2DM): a randomised controlled trial
Source: Diabetologia. 2023 Sep 29;66(12):2213–25. doi: 10.1007/s00125-023-06006-2 (PMC10627940; doi:10.1007/s00125-023-06006-2)
Supplement: Supplementary file 1 — ESM (PDF 1.25 MB) [file 125_2023_6006_MOESM1_ESM.pdf]

Supplemental material

ESM List 1: POWER2DM consortium

The following individuals participated in the design, initiation and/or completion of the POWER2DM study:

TNO:
Albert de Graaf, PhD
Maaike Beltman, PhD
Wilma Otten, PhD
Pepijn van Empelen, PhD
Shaji Krishnan, PhD
Mariël van Stee, MSc
Gino Kalkman, PhD
Eugene van Someren, PhD
Hilde van Keulen, PhD
Ton Rövekamp
Olivier Blanson Henkemans, PhD
Nicole van Kesteren, MPH
Gerrit Beumer, PhD
Jack Vogels, MSc

Leiden University Medical Center (LUMC):
Eelco J. P. de Koning, MD, PhD
Jacob K. Sont, PhD
Sasja D. Huisman, PhD
Bas S. Uitbeijerse, MD
Merel M. Ruissen, MD
Wilma S. Heemstra
Jiska Snoeck-Stroband, MD PhD
Bas Hofstee, MSc

Reina Sofia University Hospital (SAS):
Javier Delgado-Lista, MD PhD
Antonio P. Arrenas de Larriva, MD PhD
José David Torres-Peña, MD PhD
Isabel Perez Corral
Jose Carlos Prieto Baena
Juan Francisco Alcalá Díaz
Francisco José Fuentes Jiménez
Ana Isabel Pérez Caballero
Francisco Gómez Delgado
Antonio García Ríos
Ana León Acuña
Antonio Camargo Garcia
Elena Yubero Serrano
Pablo Pérez Martinez
Francisco Pérez Jiménez
José Lopez Miranda

Salzburg Research Forschungsgesellschaft m.b.H. (SRFG):
Manuela Ploessnig, MEng
Robert Mulrenin
Felix Strohmeier, DI

Oliver Jung, Beng
Dietmar Glachs, DI

Software Research & Development Consultancy (SRDC):
Tuncay Namli, PhD
Suat Gönül, PhD
Gokce Banu Laleci Erturkmen, PhD
Ozan Köse, MSc

iHealthLabs:
Armelle Merle, MEng
Uwe Diegel
Gustavo Rodriguez
Massimiliano Picone, MSc
Gustavo Serra, MBA
Louie Lyu, MSc

Institut für Diabetes Karlsburg (IDK):
Lutz Vogt, PhD
Eckhard Salzsieder, PhD
Melanie Schipfer, PhD
Kersten Rebrin, MD PhD

Primedata:
Marlies Schijf, MSc
Nielsen Vermij
Michel Sluis
Bob Donderwinkel, BSc
Roosmarijn Schopman, MSc
Mark Ekkers
Bob van der Putte

ESM Methods 1: Just In Time Adaptive Intervention Messages (JITAI).

General JITAI:

Great success! Allow yourself a small reward for it. Think of something that YOU like (a magazine/newspaper, taking a nice bath, drinking coffee on a terrace etc.)

Great job! Keep up the good work!

Excellent! You've got all your activities done and they will lead to goals!

Good work!

So fine!

That is the spirit!

You are doing great! Have you ever thought of sharing your experiences with diabetes with others? Other people may benefit from your experience! And sharing your goal progress might give an extra boost in your self-confidence. You might consider sharing your experiences in a blog.

You are making progress on your goal. Good! There might also be things that you would still like to improve. Could you think about what could you do different next week? Try to plan one specific thing for the next week, of which you think it may even further improve your progress. You can plan it on your Power2DM calendar/agenda.

You indicate that in the (#review-period) you were not in the mood for activities of goal [goal](#goal). Nice that you tell this. Do you want to know how to turn your mood in a positive direction? The following exercises can help you. Click [here](#FG-EX_MOOD) to continue. Click [here](#ACTIVITY) if you want to do it later, and schedule an activity.

You indicate in the (#review-period) that your favorite goal [goal](#goal) is currently of low importance. Would you like to have a look how important the goal is basically to you? Click [here](#FG-EX_MOTIVATION) to continue. Click [here](#ACTIVITY) if you want to do it later, and schedule an activity.

You indicate that you are not sure if you are able to perform your activities for your favorite goal [goal](#goal). Do you want to know how to increase your self-confidence? Click [here](#FG-EX_COMP) to continue. Click [here](#ACTIVITY) if you want to do it later, and schedule an activity.

Excellent! You've got all your activities for your favorite goal [goal](#goal) done. You did a great job! Now think about whether you want to continue with this goal or whether you want to change it. Click [here](#FG-EX_PLANNING-1)if you want to modify your favorite goal. Click [here](#ACTIVITY) if you want to do it later, and schedule an activity.

It seems as if you are doing less than you hoped :-(Now think about whether you want to continue with your favorite goal [goal](#goal) as it is or whether you want to change it. Click [here](#FG-EX_PLANNING-2) if you want to modify your favorite goal. Click [here](#ACTIVITY) if you want to do it later, and schedule an activity.

Well done! You are doing a great job

Keep up the good work! Hopefully the progress on your goal makes you feel more positive about yourself. Try to reflect on positive feelings about making progress on this goal.

You are doing well! Try to consciously think about how it makes you feel to achieve your goal. Do you feel proud, confident, happy, cheerful, excited? Or is there another positive emotion that you

recognize? Think about it, and try to write down all the positive feelings that you have. Even the "smallest" positive feelings are important to recognize. You can use our [well-done diary](#DIARY_journal)

Do you feel adequately informed about diabetes and its effects on you? If yes, that is important and really good! If not, you might take a look the [following information material](http://www.diabetes.co.uk/emotions/). Many people with diabetes underestimate the effect it has on them, or feel that they should not complain about it.

JITAI exercise/nutrition/glucose/medication:

Do you feel adequately informed about diabetes and its effects on you? If yes, that is important and really good! If not, you might take a look the [following information material](https://p2dm.salzburgresearch.at/). Many people with diabetes underestimate the effect it has on them, or feel that they should not complain about it.

It seems as if you are doing less than you hoped :-(Can we help you find the reason? Click here to [continue](#ODL_EX_TL)

It seems as if you are measuring your blood glucose less than you hoped :-(Can we help you find the reason? Click here to [continue](#ODL_GM_TL)

It seems as if you are doing less than you hoped :-(Can we help you find the reason? Click here to [continue](#ODL_CA).

Even small successes count! It might be good to think about what is important in your life. This is what you have said what is important for you: (#VC-key-sentence)"

Good that you are still trying! Continue your good work! Keep also in mind what is important to you in your life. This is what you have said: (#VC-key-sentence)

Good that you are still trying! Continue your good work!

Keep going! It might help to consider in which situations you have already succeeded in achieving your goals

Have you made your goal sufficiently clear and realistic so that you can reach it with reasonable effort? If not, try to reformulate your goal and your activity planning and put them into practice.

Could it be that the goal you set has proven to be unrealistic in everyday life? Then reduce your target, but keep going!

What has prevented you from exercising? What would you have to do to make it work better next week? Click here to [continue](#ODL_EX_TL)

It seems that you managed to monitor your glucose sometime this week. It might be good to list what helps you to regularly measure and record your blood glucose value. Then question yourself whether the things on your list can be realized: is your goal still achievable? Or do you think the goal or scheduling needs adjustment? If so, it is good to reformulate your goal or to set a new goal. Click [here](https://p2dm.salzburgresearch.at/adjusting-goals/) if you want to learn more on goal adjustment

Even small successes count! It might be good to think about how you can further improve on your goal next week.

Even small successes count! It might be good to think about how you can further improve on your planned activity next week.

The Value Compass helps you to become aware what is important in your life and how to life a meaningful life with your diabetes. Click here to [continue](#valuecompass)

When you feel it is difficult to achieve your goal and perform your activities, it might be good to try to remember why this goal is important to you. Can you think about the main reason(s) why you are pursuing this goal? When you have difficulty finding the importance of this goal, you might ask others (e.g. caregivers) why this goal is important in general. Then try to rethink why this goal is personally relevant to you. How can the pursuit of this goal help you in your personal life?

When you feel it is difficult to achieve your goal and perform your activities, it might be good to try to remember why this goal is important to you. Can you think about the main reason(s) why you are pursuing this goal? When you have difficulty finding the importance of this goal, you might ask others (e.g. caregivers) why this goal is important in general. Then try to rethink why this goal is personally relevant to you. How can the pursuit of this goal help you in your personal life?

Look at the [slide show](http://www.webmd.com/diabetes/controlling-diabetes-14/slideshow-control-your-blood-sugars) for controlling diabetes

Would you like to look at a quiz about diabetes? Click [here](http://www.webmd.com/diabetes/rm-quiz-diabetes-dos-donts) to continue.

Good that you are trying! What has been the most effective way to achieve this goal? You can record this in your [well-done diary](#DIARY_journal).

Good that you are trying! What has been the most effective way to perform this activity? You can record this in your [well-done diary](#DIARY_journal).

Make a note in your [well-done diary](#DIARY_journal) how you succeeded this week with your diabetes

It seems as if you are doing less than you hoped :-(Can we help you find the reason? If yes, please click [here](#PERF_OV) to continue. If you would like to do it later than schedule an [activity](#ACTIVITY).

There can be many reasons why it is hard to reach your all goals and activities. Please click on the [link](#PERF_OV) if you want to know more about why it is difficult for YOU? If you would like to do it later than schedule an [activity](#ACTIVITY).

There can be many reasons why it is hard to reach your exercise goals and activities. Please click on the [link](#PERF_EX) if you want to know more about why it is difficult for YOU?

There can be many reasons why it is hard to reach your nutrition goals and activities. Please click on the [link](#PERF_NUT) if you want to know more about why it is difficult for YOU?

There can be many reasons why it is hard to reach your glucose monitoring goals and activities. Please click on the [link](#PERF_GM) if you want to know more about why it is difficult for YOU?

There can be many reasons why it is hard to reach your medication goals and activities. Please click on the [link](#PERF_MED) if you want to know more about why it is difficult for YOU?

Maybe you would like to look at the website about [Long term complications](https://www.diabetes.co.uk/emotions/fear-of-long-term-diabetes-complications.html)

Maybe you would like to look at the website about how to cope with [lifestyle changes](https://www.diabetes.co.uk/emotions/tackling-lifestyle-changes.html)

If you dislike injections look at the following [article](http://www.diabetes.co.uk/emotions/needle-phobia.html)

If you are worried about hypoglycaemia look at the following [article](http://www.diabetes.co.uk/emotions/anxiety-over-hypoglycemia.html)

Look at the video with [exercise tips](https://www.youtube.com/watch?v=OmZ629Snyng)

Look at the video about [glucose monitoring](https://www.youtube.com/watch?v=5iq2mKoaHYA)

Look at the video with [glucose monitoring tips](https://www.youtube.com/watch?v=5iq2mKoaHYA)

It seems as if it is difficult to reach your goal. Can it be that you have set your goal too high? Often one is more successful with smaller intermediate steps. Consider whether it is useful to reduce your goal in such a way that you need to achieve the goal a bit longer, the goal is challenging for you, but still achievable in everyday life.

It seemed as if it was not your best week. That can happen. It is good that you are still trying. Remember that starting your activity after a relapse or break is really good and difficult. Try to pick up your goal with regard to (#ODL). Compliment yourself on trying again. And try not to think too negatively about the relapse/break. Relapses are part of behavior change!

It seems as if it was difficult to achieve your goal in the (#review-period). This might have been just an "off-day" or "off-week". But maybe you feel that something is hindering you in monitoring your (#ODL). It can help to discuss this with others. Do you know anybody with whom you can share questions or difficulties with regard to monitoring your (#ODL)? This can be a family member, friend, caregiver or maybe even a forum on the internet.

It seems as if it was difficult to achieve your goal in the (#review-period). This might have been just an "off-day" or "off-week". But maybe you feel that something is hindering you in monitoring your activities. It can help to discuss this with others. Do you know anybody with whom you can share questions or difficulties with regard to monitoring your goals and activities? This can be a family member, friend, caregiver or maybe even a forum on the internet.

It seems as if it was difficult to perform this activity in the (#review-period). This might have been just an "off-day" or "off-week". But maybe you feel that something is hindering you in monitoring your (#ODL). It can help to discuss this with others. Do you know anybody with whom you can share questions or difficulties with regard to monitoring your (#ODL)? This can be a family member, friend, caregiver or maybe even a forum on the internet.

Unfortunately you did not achieve your planned activity recently. There might be a very good reason why it did not work out this time. Do you know what hindered you? Do you know what you would have to do to make it work better next week? Do you want any tips for that? Click here to [continue](#ODL_EX_TL)

Unfortunately you did not achieve your planned activity recently. There might be a very good reason why it did not work out this time. Do you know what hindered you? Do you know what you would have to do to make it work better next week? Do you want any tips for that? Click here to [continue](#ODL_GM_TL)

It seems as if you did not succeed in reaching your planned activity goal this time. Do you find it difficult to turn your goals into action? Many people do so. If you want, we can provide you with some general tips on how to increase your chances of reaching your goals .If yes, please click [here](https://p2dm.salzburgresearch.at/increasing-goal-progress/) to continue. If you would like to do it later than schedule an [activity](#ACTIVITY)

It seems as if you performed less exercise than you hoped in the (#review-period). Do you know/understand why you exercised less? There can be several reasons for not performing exercise, such as not feeling like exercising, being too busy for exercise, being too tired to

exercise or feeling too much stress to exercise. We could help you in trying to change this. If you would like more information on this now, than click on [Energy Battery](#energybattery). If you would like to do the Energy Battery later than schedule an [activity](#ACTIVITY)

It seems as if you performed less exercise than you hoped. Maybe you can invite a friend or family member to join you the next time you go. Or see if you can find any local group that you could join. Doing sports together is more fun and a great opportunity to meet new people.

Look at the [slide show](http://www.webmd.com/diabetes/ss/slideshow-exercises-diabetes) with tips for best exercises if you have diabetes

Too bad you did not reach your goal! Do you know what you need to fulfill your planned sporting tasks? If you would appreciate some general tips, please click [here](https://p2dm.salzburgresearch.at/increasing-goal-progress/) to continue. If you would like to do it later than schedule an [activity](#ACTIVITY)

Using a step-counter is a good and healthy way to increase your exercise. Just wear it for 1-2 days, and look at the total number of steps. Try to increase this (average) number by 10% every few days/week. E.g. if you start with 4000 steps a day, try to step 4400 the next days/week.

Build as much movement into your everyday life as you can. Be creative! E.g. use staircases instead of the lift, try to get off a station earlier from the bus and walk the rest, make a small walk during the lunch break

On (#date) was your longest run within the (#review-period)!

It is good to measure your blood sugar. These symptoms can be an indication of either hypo-, or hyperglycaemia. If symptoms persist, please consult your doctor/nurse.

Can you ask a family member, a friend or a caregiver to remind you to take your medication regularly?

You can use the reminder function in the calendar to remind you when to take your medication

JITAI stress/personal values:

Try the [Breathing Meditation (5 mins)](http://marc.ucla.edu/mpeg/01_Breathing_Meditation.mp3) for reducing stress

Try the [Body Scan Meditation (3 mins)](http://marc.ucla.edu/mpeg/Body-Scan-Meditation.mp3) for reducing stress

Try the [Breath, Sound, Body Meditation (12 mins)](http://marc.ucla.edu/mpeg/02_Breath_Sound_Body_Meditation.mp3) for reducing stress

Look at a slide show with [relaxation tips](http://www.healthline.com/health-slideshow/10-ways-to-relieve-stress#promoSlide)

Do you know you can configure your personal relaxation tips with the [Relaxation Tip Editor](#mytips)

What do you need to relax? Think about the things that make you feel relaxed and try to plan it for (later) today.

Can you plan more breaks? Even a small 1-5 min. break can be very effective in reducing stress. Try to plan a small break every hour.

It seems your stress level was quite high in the (#review-period). Could it be that some areas in your life are in conflict with your diabetes? Can we help to find the reasons? The [Value Compass](#valuecompass) is an exercise helping you to reconstruct the balance between your health goals and your personal goals.

It seems your stress level was quite high in the last month. Could it be that some areas in your life are in conflict with your diabetes? Can we help to find the reasons? The [Value Compass](#valuecompass) is an exercise helping you to reconstruct the balance between your health goals and your personal goals.

It seems your stress level was quite high in the (#review-period). We have an exercise to help you reduce your stress and improve your mood. If you would like to continue with the exercise now than click on [Energy Battery](#energybattery). If you would like to do the Energy Battery later than schedule an [activity](#ACTIVITY)

It seems your stress level was quite high in the last month. We have an exercise to help you reduce your stress and improve your mood. If you would like to continue with the exercise now than click on [Energy Battery](#energybattery). If you would like to do the Energy Battery later than schedule an [activity](#ACTIVITY)

We noticed you had a lot of stress. Would you like to become more aware of early warning symptoms before you are stressed out? Please click [here](https://p2dm.salzburgresearch.at/traffic-light/) to continue. If you would like to do the exercise later than schedule an [activity](#ACTIVITY)

What do you need to relax? This is what you have said: (#relaxation-tip)"

We noticed you had a lot of stress. You can choose in your leisure time activities that don’t look like work activities, e.g. (#relaxation-tip)

Think how you would react if someone asked for support.

It is not always clear for your family and friends that you need support. In that case you need to ask for it. If you find this hard to do, then think how you would respond if someone asks for your help. And: you can better ask for help than completely fail."

Seek support from family and/or friends.

You experience support when you are taken seriously, when people listen to you, and when they care about you. Support can also be practical: someone who thinks with you about a problem, or someone who advises how to solve a problem."

Plan moments to relax.

A busy private life and doing many fun things or each evening watching TV, are both no guarantees for relaxation. Plan time in your agenda to do things that help you to relax. Which activities clear your mind and distract your thoughts?"

Choose in your leisure time activities that don’t look like work activities.

Even though you fulfill your work and private obligations, then it is still important to take time to relax and lower your stress level."

"In your leisure time try to plan activities that energize you.

Manage your spare time and take care to spend time to do activities that refresh and energize you. "

Critically review your private activities.

Are all those chores on your list really necessary? Do you have to do them? Is it possible to get help?

JITAI weight:

If you are gaining weight with your diabetic therapy, if would be good to discuss that with your healthcare professional (eg doctor, dietician).

Thanks for sharing this. That is good to know. You are not alone. Many people with diabetes struggle with the fear of gaining weight. Click [here](https://p2dm.salzburgresearch.at/struggling-with-the-fear-of-gaining-weight/) if you want to know more about the fear of gaining weight. Click [here](#ACTIVITY), if you want to do it later and schedule an activity.

JITAI sleep:

Try to make sure you get adequate sleep. Most adults need 7-8 hours of sleep to feel refreshed in the morning. Try changing your bedtime, by going to bed 15 minutes earlier (every few days/week) until you feel rested in the morning.

Try to always go to bed at the same time and get up at the same time, even on the weekends.

Noise and light may hinder your sleep. If this might be a problem for you, try earplugs and a sleeping mask or buy dark curtains

Room temperature is an important factor in sleep quality. Keep your bedroom cool and well-ventilated.

To make sure your brain is stimulated to sleep, try to declare your bedroom to a "sleeping zone," without computer, TV and other work-related items.

To ensure good sleep, our brain needs to be "relaxed" when going to bed. Try to do something relaxing before you go to sleep, like taking a warm bath, reading a pleasurable book, listening to soft music or meditation

By eating and drinking your body and brain are stimulated, which may hinder your sleep. Try to avoid eating and drinking just before going to bed. When possible, don't eat larger meals 2-3 hours before going to bed. Do not drink too much before going to bed. This avoids nightly toilets.

Research shows that regular exercise improves your sleep quality. 30 minutes of daily exercise improves our sleep. But try avoid any exercise within 3 hours before going to sleep.

Certain foods and drinks have a stimulating effect. Try to avoid caffeine (coffee, tea, cola, energy drinks, dark chocolate) after noon.

To increase your sleep quality, it is best to avoid naps during the day. If you need one, try to do it before 15:00 and not sleep longer than 30 minutes. Use an alarm to make sure you will stick to the 30 minutes.

Some medications have a sleep-inhibitory effect, discuss with your doctor / your prescriber whether one of your drugs can interfere with your sleep.

It seems your sleep quality was a bit low in the (#review-period). We have an exercise to help you improve your sleep quality. Would you like to do it now? If yes, than click on [Energy Battery](#energybattery). If you would like to do the Energy Battery later than schedule an [activity](#ACTIVITY)

It seems your sleep quality was a bit low in the last month. We have an exercise to help you improve your sleep quality. Would you like to do it now? If yes, then click on [Energy Battery](#energybattery). If you would like to do the Energy Battery later than schedule an [activity](#ACTIVITY)

Do you know that nicotine has a stimulating effect? Therefore, it is best not to smoke just before going to sleep.

JITAI mood:

It seems your mood was a bit low in the (#review-period). We have an exercise to help you improve your mood. Would you like to do it now? If yes, then click on [Energy Battery](#energybattery). If you would like to do the Energy Battery later than schedule an [activity](#ACTIVITY)

It seems your mood was a bit low in the last month. We have an exercise to help you improve your mood. Would you like to do it now? If yes, then click on [Energy Battery](#energybattery). If you would like to do the Energy Battery later than schedule an [activity](#ACTIVITY)

It seems your mood was a bit low in the (#review-period). Could it be that some areas in your life are in conflict with your diabetes? Can we help to find the reasons? The [Value Compass](#valuecompass) is an exercise helping you to reconstruct the balance between your health goals and your personal goals.

It seems your mood was a bit low in the last month. Could it be that some areas in your life are in conflict with your diabetes? Can we help to find the reasons? The [Value Compass](#valuecompass) is an exercise helping you to reconstruct the balance between your health goals and your personal goals.

We noticed that your mood was low. Would you like to become more aware of early warning symptoms before your mood becomes low? Please click [here](https://p2dm.salzburgresearch.at/traffic-light/) to continue. If you would like to do the exercise later than schedule an [activity](#ACTIVITY)

We noticed that your mood was low in the last month. Would you like to become more aware of early warning symptoms before your mood becomes low? Please click [here](https://p2dm.salzburgresearch.at/traffic-light/) to continue. If you would like to do the exercise later than schedule an [activity](#ACTIVITY)

JITAI general problems:

We would advise you to mention these symptoms in the next appointment/telephone call with your diabetes nurse or doctor. If the symptoms persevere or (rapidly) get worse, contact a doctor for advice.

Sexual problems are more common in people with diabetes. Both men and women may experience symptoms because of their diabetes that influence their sexual wellbeing. Click [here](https://p2dm.salzburgresearch.at/experiencing-diabetes/daily-life-with-diabetes/sexual-activity/) if you want to read more about these symptoms. Click [here](#ACTIVITY) if you want to do it later, and schedule an activity.

It seems you felt weak, tired and/or powerless in the (#review-period). We have an exercise to help you to cope with that. Would you like to do it now? If yes, then click on [Energy

Battery](#energybattery). If you would like to do the Energy Battery later than schedule an [activity](#ACTIVITY)

It seems you felt weak, tired and/or powerless in the last month. We have an exercise to help you to cope with that. Would you like to do it now? If yes, then click on [Energy Battery](#energybattery). If you would like to do the Energy Battery later than schedule an [activity](#ACTIVITY)

JITAI glucose/self-management/psychological burden:

It seems as if you checked your glucose values less than you hoped in the (#review-period). Could it be that some areas in your life are in conflict with your diabetes? Can we help to find the reasons? The [Value Compass](#valuecompass) is an exercise helping you to reconstruct the balance between your health goals and your personal goals.

It seems as if you checked your glucose values less than you hoped in the last month. Could it be that some areas in your life are in conflict with your diabetes? Can we help to find the reasons? The [Value Compass](#valuecompass) is an exercise helping you to reconstruct the balance between your health goals and your personal goals.

Did you feel any symptoms preceding your hypo(s)? If not and if you would like to learn, we have an exercise to help you to cope with that. Please click [here](https://p2dm.salzburgresearch.at/traffic-light/). If you would like to do the Traffic Light later than schedule an [activity](#ACTIVITY)

Could it be that some areas in your life are in conflict with your diabetes? Can we help to find the reasons? The [Value Compass](#valuecompass) is an exercise helping you to reconstruct the balance between your health goals and your personal goals

Most people are aware of the dangers of persistent high glucose levels, but do not ruminate (too much) about them. However, some people worry about diabetes complications more than is good/healthy for them. Click [here](https://p2dm.salzburgresearch.at/about-diabetes-complications/) if you want to read more about fear of complications. Click [here](#ACTIVITY) if you want to do it later, and schedule an activity.

Good to know. Many people with diabetes worry about having low blood sugar (hypoglycaemia, or ‘hypo’s’). Click [here](https://p2dm.salzburgresearch.at/?p=1136&preview=true) if you want to read more about fear of hypoglycaemia. Click [here](#ACTIVITY) if you want to do it later, and schedule an activity.

You may have used the tips and advices we gave when you recognized your fear of hypoglycaemia and your excessive safety behaviors. If you want to work on limiting the excessive safety behaviors yourself click [here](https://p2dm.salzburgresearch.at/coping-with-your-fear-of-hypoglycaemia/). If you want to do it later, schedule an [activity](#ACTIVITY).

You may have used the tips and advices we gave when you recognized your fear of hypoglycaemia and your excessive safety behaviors. If you want to work on limiting the excessive safety behaviors yourself click [here](https://p2dm.salzburgresearch.at/coping-with-your-fear-of-hypoglycaemia/). If you want to do it later, schedule an [activity](#ACTIVITY).

Sometimes, negative emotions are not directly related to blood sugar, but more indirectly related to diabetes: people may be dissatisfied with their self-management, feel frustrated/guilty about high/low blood sugar, experience a lack of understanding in people around them. Click [here](https://p2dm.salzburgresearch.at/more-about-negative-emotions/) if you want to read more about it. Click [here](#ACTIVITY) if you want to do it later and schedule an activity.

We know that many people with diabetes experience negative emotions because of their diabetes. You mentioned, that doing exercise gives you negative emotions. Click [here](https://p2dm.salzburgresearch.at/coping-with-negative-emotions-because-of-exercise/) if you want to read more on negative emotions about exercise. Click [here](#ACTIVITY) if you want to do it later and schedule an activity.

You reported to have negative emotions when (not) monitoring your blood glucose. Click [here](https://p2dm.salzburgresearch.at/emotion-monitoring/) if you want to read more about negative emotions and glucose monitoring. Click [here](#ACTIVITY). if you want to do it later, and schedule an activity.

We know that many people with diabetes experience negative emotions because of their diabetes. You mentioned, that monitoring your glucose gives you negative emotions. Click [here](https://p2dm.salzburgresearch.at/about-negative-emotions-because-of-your-diabetes/) if you want to read more on negative emotions in glucose monitoring. Click [here](#ACTIVITY) if you want to do it later, and schedule an activity.

Sometimes, negative emotions are not directly related to blood sugar, but more indirectly related to diabetes: Click [here](https://p2dm.salzburgresearch.at/more-about-negative-emotions/) if you want to read more about it. Click [here](#ACTIVITY) if you want to do it later, and schedule an activity.

You reported to have negative emotions when (not) monitoring your blood glucose. Click [here](https://p2dm.salzburgresearch.at/emotion-monitoring/) if you want to read more about negative emotions and glucose monitoring. Click [here](#ACTIVITY) if you want to do it later, and schedule an activity .

Sometimes, negative emotions are not directly related to blood sugar, but more indirectly related to diabetes: Click [here](https://p2dm.salzburgresearch.at/more-about-negative-emotions/) if you want to read more about it. Click [here](#ACTIVITY) if you want to do it later, and schedule an activity.

We know that many people with diabetes experience negative emotions because of their diabetes. You mentioned, that monitoring your glucose gives you negative emotions. Click [here](https://p2dm.salzburgresearch.at/about-negative-emotions-because-of-your-diabetes/) if you want to read more on negative emotions in glucose monitoring. Click [here](#ACTIVITY) if you want to do it later, and schedule an activity.

Some people with diabetes feel uncomfortable telling about their diabetes, or about performing their self-management in public. If you recognize this, and want to read more about it, click here [here](https://p2dm.salzburgresearch.at/fear-disclosure/). If you want to do it later, and schedule an activity, click [here](#ACTIVITY) .

In daily practice, many people with diabetes say that they 'forget' about specific parts of diabetes self-management. Click [here](https://p2dm.salzburgresearch.at/forgetting-about-diabetes-self-management/) if you want to read more about forgetting (parts of) your diabetes self-management and what to do about it. Click [here](#ACTIVITY) if you want to do it later, and schedule an activity .

Sometimes, people with diabetes try their hardest without any apparent result… Of course, this might lead to feelings of frustration. Click [here](https://p2dm.salzburgresearch.at/how-to-deal-with-frustration/) if you want to read more about it. Click [here](#ACTIVITY) if you want to do it later and schedule an activity.

When not fulfilling/reaching our goals, many people start 'blaming' themselves and (unconsciously) start negative self-talk. Stopping this negative self-talk is not easy, but really

necessary when trying to change behavior. Click [here](https://p2dm.salzburgresearch.at/negative-self-talk/) if you want to read more. Click [here](#ACTIVITY) if you want to do it later, and plan an activity.

When not fulfilling/reaching our goals, many people start ‘blaming’ themselves and (unconsciously) start negative self-talk. Stopping this negative self-talk is not easy, but really necessary when trying to change behavior. Click [here]https://p2dm.salzburgresearch.at/coping-with-negative-self-talk/) if you want to do an exercise on this. Click [here](#ACTIVITY) if you want to do it later, and plan an activity.

Thanks for sharing how you feel. If you feel exercising is not (so) important to you, we understand that it is difficult to perform (more) exercise. Click [here](https://p2dm.salzburgresearch.at/why-exercises-are-important/) if you want to read how you could try to increase the personal relevance of doing exercise. Click [here](#ACTIVITY) if you want to do it later, and schedule an activity.

Thanks for sharing how you feel. We can understand that it might be difficult to see how doing more exercise could help you in improving your health. The good news is: exercise really DOES improve your health!! Click [here](https://p2dm.salzburgresearch.at/importance-of-exercise/) if you want to read more about it. Click [here](#ACTIVITY) if you want to do it later, and schedule an activity.

We know that many people with diabetes experience negative emotions because of their diabetes. You mentioned, that doing exercise gives you negative emotions. Click [here](https://p2dm.salzburgresearch.at/coping-with-negative-emotions-because-of-exercise/) if you want to read more on negative emotions about exercise. Click [here](#ACTIVITY) if you want to do it later and schedule an activity.

Sometimes, negative emotions are not directly related to blood sugar, but more indirectly related to diabetes: people may be dissatisfied with their self-management, feel frustrated/guilty about high/low blood sugar, experience a lack of understanding in people around them. Click [here](https://p2dm.salzburgresearch.at/more-about-negative-emotions/) if you want to read more about it. Click [here](#ACTIVITY) if you want to do it later and schedule an activity.

Fear of needles is a common, but very hindering phenomenon in people with diabetes. Click here [here](https://p2dm.salzburgresearch.at/fear-of-needles/) if you want to read more about fear of needles and diabetes. Click [here](#ACTIVITY) if you want to do it later, and schedule an activity.

When you feel anxious about having to inject, it is good to follow a stepwise plan to ‘’practice’’ with injecting, and trying to overcome your fears. If you want to continue with an exercise about fear of needles, click [here](https://p2dm.salzburgresearch.at/coping-with-fear-of-needles/). Click [here](#ACTIVITY) if you want to do it later, and schedule an activity. That will remind you.

Thanks for sharing how you feel. If monitoring is not (so) important to you, we understand that it is difficult to keep monitoring. Click [here](https://p2dm.salzburgresearch.at/increasing-the-personal-relevance-of-monitoring/) if you want to read how you could try to increase the personal relevance of monitoring. Click [here](#ACTIVITY) if you want to do it later, and schedule an activity.

Good to know that you question the usefulness of monitoring your glucose levels. We rather know, and try to help you. Click [here](https://p2dm.salzburgresearch.at/importance-of-glucose-monitoring/) if you want to read more on the importance of glucose monitoring. Click [here](#ACTIVITY) if you want to do it later, and schedule an activity.

Thanks for sharing how you feel. If you monitoring is not (so) important to you, we understand that you are not happy about. Click [here](https://p2dm.salzburgresearch.at/increasing-the-personal-relevance-of-monitoring/) if you want to read how you could try to increase the personal relevance of monitoring. Click [here](#ACTIVITY) if you want to do it later, and schedule an activity.

It is good that you reported this! Not knowing how or when to assess your blood sugar is an important barrier in your diabetes self-management. We encourage you to contact your health care professional. Click [here](https://p2dm.salzburgresearch.at/when-you-dont-know-how-or-when-to-assess-your-blood-sugar/) if you want to read a little more. Click [here](#ACTIVITY) if you want to do it later, and schedule an activity.

Sorry that we couldn't help you by giving more information. Please make note of your question or remark. It may be good to share it with your health professional in the next consultation.

Good that you mention this! Optimal and comfortable self-management is all about having the knowledge and skills. If you feel that you lack knowledge or skills in a specific area, it is good to tell your health care professional. He/she is probably happy to tell/teach you. Don't be afraid or ashamed to ask your questions or even to repeat them. This is important!

Sometimes pursuing a goal can be frustrating. A little frustration every now and then might be unpleasant, but is usually a sign that you are trying something 'new' or 'different'. This new behavior is not automatic behavior yet, and will cost you more energy (and frustration) than the old behavior. Try to remember this when feeling a little frustrated: you are making progress! If you feel that your frustrations are too hindering in pursuing your goal, click [here](https://p2dm.salzburgresearch.at/adjusting-goals/) to read more on how you might adjust your goal.

Being active is important not only for your health, but also for your mind. Research shows that physical exercise and sports help to improve low mood, or even depressions. However, when feeling depressed most people find it difficult to perform exercise or sports. They feel they lack the energy to do it. If you recognize this, you might be interested in watching this [video](media/energy_battery.mp4) on low energy/mood. If you want to read more about depression in general, you can click [here](https://p2dm.salzburgresearch.at/diabetes-and-depression/)

Being active is important not only for your health, but also for your mind. Research shows that physical exercise and sports help to improve low mood, or even depressions. However, when feeling depressed most people find it difficult to perform exercise or sports. They feel they lack the energy to do it. If you recognize this, you might be interested in watching this [video](media/energy_battery.mp4) on low energy/mood. If you want to read more about depression in general, you can click [here](https://p2dm.salzburgresearch.at/diabetes-and-depression/)

Good to know that you question the importance of your goal. We rather know and help you with it. Pursuing a goal that does not feel important to you is a nuisance (negative emotion). Next to that, our brain has less attention for things that are not important to us, so it is hard to perform a behavior with less attention AND a negative emotion.

When you question the importance of your goal, it would be good to discuss it with a health care provider. Maybe the relevance has not been explained yet, and you could benefit from a better explanation of why the pursuit of this specific goal is thought to be beneficial to you.

Or else, it would be good to adjust the goal. If you want to know more on goal adjustment, click [here](https://p2dm.salzburgresearch.at/adjusting-goals/).

Click [here](#ACTIVITY) if you want to do it later, and schedule an activity.

In psychology we use the metaphor of a traffic light to explain how our positive feelings and sensations can turn into really negative feelings or sensations. This traffic light metaphor explains how we ‘’suddenly’’ become angry, panicked, sad, depressed. But it also helps to understand how your blood sugar ‘’suddenly’’ may rise or drop. Click

[here](https://p2dm.salzburgresearch.at/traffic-light/) if you want to read more. Click [here](#ACTIVITY) if you want to do it later and schedule an activity.

Being active, doing things we like helps to maintain or improve our well-being. If you want to engage more in pleasurable activities, but you are unsure what to do, click [here](https://p2dm.salzburgresearch.at/pleasurable-activities/). Click [here](#ACTIVITY) if you want to do it later and schedule an activity.

When you are not making progress in your goal(s), it is good to reflect on what is happening. It might be good to revise and adjust your goals. Click [here](https://p2dm.salzburgresearch.at/adjusting-goals/) if you want to read more on the importance of goal adjustment. Click [here](#ACTIVITY) if you want to do it later and schedule an activity.

Self-managing your diabetes can be quite a challenge. Many people with diabetes find it difficult to combine their 'diabetes goals' with all the other things they do in daily life. Click [here](https://p2dm.salzburgresearch.at/combing-your-diabetes-goals-with-the-other-things-in-your-daily-life/) if you want to read more on how to create more balance between your diabetes goals and other, more personal goals. Click [here](#ACTIVITY) if you want to do it later and schedule an activity.

Stress will always be a part of our lives. No matter how smooth and efficient we organize our jobs and private lives, stress will sometimes be part of it. Still, there are some general tips that may help in preventing too much stress. Click [here](https://p2dm.salzburgresearch.at/stress-prevention/) if you want to read more on stress prevention. Click [here](#ACTIVITY) if you want to do it later and schedule an activity.

Stress and diabetes have a complex relationship. It is well known that stress influences your diabetes, in both direct and indirect ways. Click [here](https://p2dm.salzburgresearch.at/coping-with-your-stress/) if you want to read more on diabetes and stress. Click [here](#ACTIVITY) if you want to do it later and schedule an activity.

When not having reached your goal, or not having succeeded in following upon your plans, there might be many reasons and explanations. Click [here](https://p2dm.salzburgresearch.at/increasing-goal-progress/) if you want some general tips to increase your chances of goal progress. Click [here](#ACTIVITY) if you want to do it later and schedule an activity.

Knowing that lifestyle is an important factor in the development of type 2 diabetes, many people incorrectly assume that having diabetes ''is their own fault'', or that ''they are to blame''. But having feelings of guilt or self-blame is not helpful. In fact, it can even bring you in a negative vicious cycle. Click [here](https://p2dm.salzburgresearch.at/if-yopu-have-feelings-of-guilt/) if you want to learn more about how to change these feelings. Click [here](#ACTIVITY) if you want to do it later and schedule an activity.

Depression can be diagnosed by a health care professional, based on specific symptoms. If you want to read more about these specific symptoms, click [here](https://p2dm.salzburgresearch.at/symptoms-of-depression/) if you want to read more. Click [here](#ACTIVITY) if you want to do it later and schedule an activity.

Research shows that people with diabetes are twice as likely to suffer from depression. Click [here](https://p2dm.salzburgresearch.at/diabetes-and-depression/) if you want to read more about diabetes and depression. Click [here](#ACTIVITY) if you want to do it later, and schedule an activity.

We know that many people with diabetes experience negative emotions because of their diabetes. You mentioned, that taking your diabetes medication (either tablets or insulin) gives you negative emotions. Click [here](https://p2dm.salzburgresearch.at/negative-emotions-because-of-your-medication/) if you want to read more on negative emotions about diabetes medication. Click [here](#ACTIVITY) if you want to do it later and schedule an activity.

It is good that you reported this! Not knowing how to monitor or reduce your carbohydrates is an important barrier in your diabetes self-management. We encourage you to contact your health care professional. Click [here](https://p2dm.salzburgresearch.at/monitoring-your-carbohydrates/) if you want to read a little more. Click [here](#ACTIVITY) if you want to do it later and schedule an activity.

ESM Methods 2: Inclusion and exclusion criteria POWER2DM study.

Inclusion criteria

To be eligible to participate in this study, a subject must meet all of the following criteria:

• Age 18 or older

• Diagnosed T2DM or T1DM

• Able to self-monitor and work with computer and smart phone with internet connections (as assessed by researcher)

Exclusion criteria

A potential subject who meets any of the following criteria will be excluded from participation in this study:

• Severe renal insufficiency (eGFR<30ml/min)

• Serious/severe comorbidity that interferes with diabetes outcomes or diabetes self-management including but not limited to: psychiatric diseases, chronic hepatopathy, active malignancy, COPD, diseases of the digestive tract, endocrine disorders, cerebrovascular disease with disability

• For female participants: pregnancy or wanting to become pregnant in the coming 9 months

• Concurrent participation in other clinical trials

• Any other situation in which the investigator identifies a potential risk of not being able to perform the study.

ESM Methods 3: Primary and secondary outcomes.

Description of outcomes

In venous blood samples HbA_{1c}, total cholesterol, HDL cholesterol, LDL cholesterol and triglycerides were measured. Anthropometrics consisted of height and body weight. Data on ethnicity and race were not analyzed, since participants were predominantly Caucasian. collected. Blood pressure measurements were performed at each visit. To assess potential barriers for self-management patients in the POWER2DM group completed questionnaires on diabetes distress (Problem Areas In Diabetes questionnaire (PAID))[1], fear of injections / fear of needles (Diabetes Fear of Injecting and Self-Testing Questionnaire (D-FISQ)[2]), fear of hypoglycaemic episodes (Clarke Hypoglycemia Unawareness Instrument)[3], Hypoglycaemic Fear Survey II (HFS II)[4]) and fear of complications (Fear of Complications Questionnaire (FCQ)[5]). To assess the overall effect of POWER2DM integrated e-health on quality of life and diabetes self-management all patients completed the WHO wellbeing index (WHO-5) and the Diabetes Self-Management Questionnaire (DSMQ-R). Furthermore, patients randomized to the POWER2DM group completed the technology acceptance questionnaire (TAQ)[6] (ESM Questionnaire 1) at the end of the study. Data from unblinded intermittently scanned continuous glucose monitoring devices (regular FreeStyle Libre) of patients in the POWER2DM group were not used for data analysis. Outcomes of blinded continuous glucose monitoring devices (FreeStyle Libre Pro) were defined as percentage of time <3.0 mmol/L (<54 mg/dL), percentage of time between 3.0-3.8 mmol/L (54-69 mg/dL), percentage of time in range: 3.9-10.0 mmol/L (70-180 mg/dL), percentage of time between 10.1 and 13.9 mmol/L (181-250 mg/dL) and percentage of time >13.9 mmol/L (>250 mg/dL).[7]

Complete list of secondary outcomes (as registered on clinicaltrials.gov) and considerations concerning data selection.

1. Amount hypoglycaemia [0 weeks, 11 weeks, 22 weeks and 37 weeks]

Amount of hypoglycaemia measured by time spent in hypoglycaemia before and after treatment in the Power2DM group compared to the usual care control group

2. Hypo unawareness [0 weeks, 11 weeks, 22 weeks and 37 weeks]

Hypo unawareness as measured by Clarke hypoglycaemia unawareness instrument, before and after treatment in the Power2DM group compared to the usual care control group

3. Incidence of adverse events [0 weeks, 11 weeks, 22 weeks and 37 weeks]

Incidence of adverse events occurring during the study period including serious hypoglycaemic events

4. Mean blood glucose (MBG) [0 weeks, 11 weeks, 22 weeks and 37 weeks]

As derived from continuous glucose measurements made by (intermittently scanned) continuous glucose monitoring devices

5. Standard deviation of MBG (SDBG) [0 weeks, 11 weeks, 22 weeks and 37 weeks]

As derived from continuous glucose measurements made by (intermittently scanned) continuous glucose monitoring devices

6. Largest amplitude of glycaemic excursions (LAGE) [0 weeks, 11 weeks, 22 weeks and 37 weeks]

As derived from continuous glucose measurements made by (intermittently scanned) continuous glucose monitoring devices

7. Mean amplitude of glycaemic excursions (MAGE) [0 weeks, 11 weeks, 22 weeks and 37 weeks]

As derived from continuous glucose measurements made by (intermittently scanned) continuous glucose monitoring devices

8. Absolute means of daily differences (MODD) [0 weeks, 11 weeks, 22 weeks and 37 weeks]

As derived from continuous glucose measurements made by (intermittently scanned) continuous glucose monitoring devices

9. Time spent in range [0 weeks, 11 weeks, 22 weeks and 37 weeks]

As derived from continuous glucose measurements made by (intermittently scanned) continuous glucose monitoring devices

10. ADVANCE Cardiovascular risk [0 weeks, 11 weeks, 22 weeks and 37 weeks]

The ADVANCE Cardiovascular Risk Engine, calculates the risk of major cardiovascular disease in patients with type 2 diabetes for the next 4 years (range 0-100%). This is defined as fatal or non-fatal myocardial infarction, stroke or cardiovascular death.

11. ADVANCE Kidney disease Risk [0 weeks, 11 weeks, 22 weeks and 37 weeks]

The ADVANCE Kidney Risk Engine, calculates the risk of new-onset albuminuria and major kidney-related events in patients with type 2 diabetes for the next 5 years (range 0-100%). Major kidney-related events are defined as doubling of serum creatinin to >2.26mg/dL, renal replacement therapy, or renal death.

12. Major Outcomes T1D [0 weeks, 11 weeks, 22 weeks and 37 weeks]

The Major Outcomes T1D risk score assess the 3, 5 and 7 year risk of a patient with type 1 diabetes on major outcomes (range 0-100%). These outcomes included major coronary heart disease, stroke, end-stage renal failure, amputations, blindness and all-cause death.

13. UKPDS risk score [0 weeks, 11 weeks, 22 weeks and 37 weeks]

The UKPDS risk score calculated the risk a patient with type 2 diabetes will develop coronary heart disease, fatal coronary heart disease, stroke or fatal stroke (range 0-100%)

14. Q score [0 weeks, 11 weeks, 22 weeks and 37 weeks]

The Q score is a single metric for a continuous glucose monitoring (CGM) profile which summarizes the glucose profile using five factors: central tendency, hyperglycaemia, hypoglycaemia, intra- and inter daily variations.

15. Amount of steps [0 weeks, 11 weeks, 22 weeks and 37 weeks]

Average amount of steps per day over a week measured by a step counter

16. Self-reported exercise time [0 weeks, 11 weeks, 22 weeks and 37 weeks]

Exercise time per week as reported in the POWER2DM system

17. Frequency of self-monitoring of blood glucose (SMBG) measurements [0 weeks, 11 weeks, 22 weeks and 37 weeks]

Frequency of SMBG measurements as reported by the glucose measurement device

18. Self-reported adherence to medication plan [0 weeks, 11 weeks, 22 weeks and 37 weeks]

Self-reported adherence to medication plan as reported in the POWER2DM system

19. Weight [0 weeks, 11 weeks, 22 weeks and 37 weeks]

Weight in kilograms measured on a scale

20. Body mass index (BMI) [0 weeks, 11 weeks, 22 weeks and 37 weeks]

BMI in kg/m2, computed from height and weight

21. Diabetes Self-Management Questionnaire Revised (DSMQ-R) [0 weeks, 11 weeks, 22 weeks and 37 weeks]
Subscales: Glucose Management, Dietary Control, Physical Activity, Health Care Use
Transformed scale scores can vary between 0-10, with higher scores indicating more effective self-care

22. Patient utilities by EQ-5D [0 weeks, 11 weeks, 22 weeks and 37 weeks]
No subscales: EQ-5D provides a general health index with higher scores indicating better general health. QALYs will be calculated from EQ-5D scores.

23. Problem Areas in Diabetes (PAID) [0 weeks, 11 weeks, 22 weeks and 37 weeks]
The PAID provides a total diabetes distress score (0-100), with higher scores (> 40) indicating more distress.

24. Mood/Well-being by WHO-5 and Patient Health Questionnaire (PHQ-9) [0 weeks, 11 weeks, 22 weeks and 37 weeks]
WHO-5 provides a total score (0-100) with higher scores indicating better wellbeing, PHQ-9 provides a total score (1-27) indicating a likelihood of depression, with higher scores indicating more depressive symptoms

25. Technology Acceptance Questionnaire (TAQ) [5 weeks and 37 weeks]
The TAQ provides scores (1-7) on the following domains: Performance expectancy, Effort expectancy, Social influence, Facilitating conditions, Affect, Self-efficacy, Trust, Motivation and Behavioural intention. Higher scores indicate better acceptance of the system.

26. Cost-effectiveness [Over 37 weeks]
Costs/quality adjusted life years (QALYs) Costs assessed via cost questionnaire and medication registry. QALYs based on patient utilities measured via EQ5D.

27. Stress by perceived Stress Scale (PSS) [0 weeks, 11 weeks, 22 weeks and 37 weeks]
The PSS provides a total perceived stress score (0-40), with higher scores indicating more perceived stress.

28. Patient Assessment of Chronic Illness Care (PACIC) [0 weeks, 11 weeks, 22 weeks and 37 weeks]
The PACIC measures the patient's perception of the care that they receive.

The current manuscript focuses only on the effect of POWER2DM on glycaemic control, diabetes self-management and quality of life and therefore does only report on a selection of secondary outcomes (1, 2, 3, 9, 19, 20, 21, 24 and 25).

ESM Methods 4: Detailed description of statistical analyses.

Sample size and power calculations

Sample size requirements were calculated based on a minimal detectable difference of 0.35% (SD 0.9%) (3.8 mmol/mol (SD 9.8 mmol/mol)) in the primary outcome variable HbA_{1c}. For an alpha error of 0.05 and a power of 80%, the minimum sample size needed was 104 subjects per group. Therefore, we aimed to include a total of 115 patients with type 1 diabetes and 115 patients with type 2 diabetes. This allowed us to face a loss to follow-up of up to 9.6%. In pre-specified subgroup analyses of patients with type 1 and type 2 diabetes a difference of 0.5% (5.5 mmol/mol) in HbA_{1c} could be detected with a sample size of 51 subjects per treatment strategy per diabetes subtype (N=57 with 11% loss to follow-up).

Statistical analyses

All outcomes were analysed using the STATA xtmixed command for multi-level linear regression. Visual inspection of outcome graphs suggested a stable intervention effect in the most important outcomes, already after 3 months. Therefore, we used the same approach for all (continuous) endpoints, by including indicators for follow-up period (3-9 months) and randomization group as fixed factors into the model to adjust for baseline differences and random factors to allow adjustment for repeated measurements within a patient (see figure below).

Mixed-model analysis

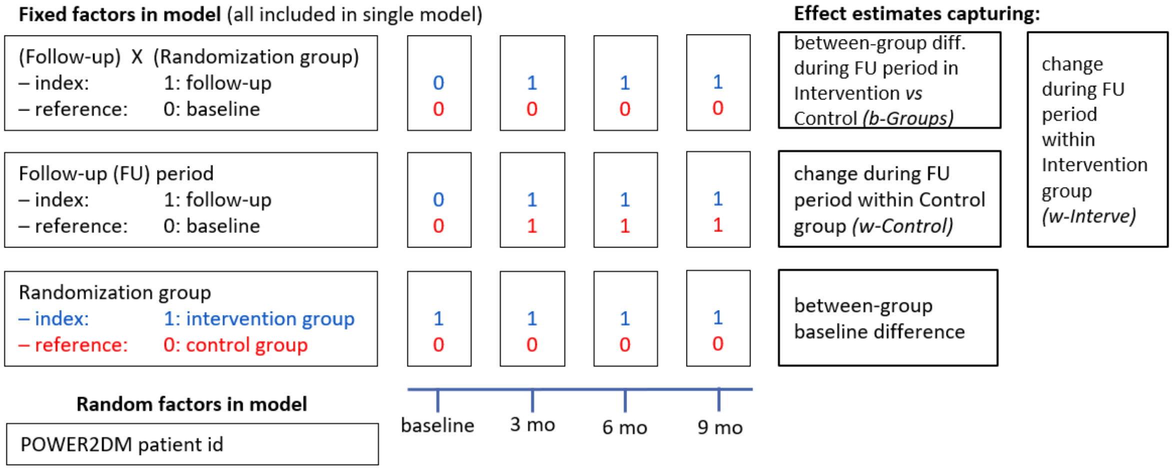

An interaction term (randomization group_X_follow-up) was included in the model to assess possible differences in outcomes between the groups during the follow-up period. For all endpoints we performed an overall analysis of all patients (patients with type 1 diabetes and type 2 diabetes combined) and two separate analyses for patients with type 1 and type 2 diabetes.
Despite not being explicitly stated on the clinicaltrials.gov website, subgroup analyses in patients with type 1 and type 2 diabetes were pre-planned. See POWER2DM deliverable D5.2.2 Evaluation of Campaign Methodology (https://www.power2dm.eu/wp-content/uploads/Power2DM-D5.3.pdf), page 19.

We used multiple imputation by chained equations (MICE) which uses a separate conditional distribution and model for each imputed variable and allows imputation of outcome data at a specific visit by including (imputed) data obtained at other visits[8]. Before running MICE in STATA the data were reshaped from long format (one observation per patient per record) to wide format (a single record per patient). Missing values in the dependent variable were fitted by linear regression using all available measurements of the respective outcome at other timepoints in addition to diabetes type, center, sex, randomization group and treatment. Since MICE is an iterative process, the variable with the fewest missing values is imputed first followed by the variable with the next fewest missing values and so on for the rest of the variables. We used the default number of five datasets to be imputed. We used a random seed number (9478) in order to obtain reproducible results. After MICE this reshape procedure was reversed to obtain five imputed datasets in long format with an indicator variable for imputation set.

Most multiple imputation assumes that the data come from a multivariate normal distribution, however, the procedures are robust to moderate deviation from normality in typically sized trials[9]. We therefore did not check convergence, but did check whether imputed data were within the plausible range.

ESM Technology Acceptance Questionnaire (TAQ)

We want to ask you a few questions about your current view on the Power2DM system and your expectations.
With the Power2DM system we mean the app, the web application and devices with the different applications to support your self-management.
For us it is important to know how you think about it at the moment. There are no right or wrong answers, it is your opinion.

The first question is about your motivation for using Power2DM. Please indicate to what extent you agree with the statement.

I am motivated to continue using the Power2DM system.

( ) Completely disagree

( ) Mostly disagree

( ) Somewhat disagree

( ) Neither agree nor disagree

( ) Somewhat disagree

( ) Mostly agree

( ) Completely agree

( ) Not applicable

There are several reasons for continuing to work with the Power2DM system. Below are a number of reasons. To what extent do they apply to you?

"I want to continue working with the Power2DM system, because ....

	**Completely disagree**	**Mostly disagree**	**Somewhat disagree**	**Neither agree nor disagree**	**Somewhat agree**	**Mostly agree**	**Completely agree**	**Not applicable**
I find it a personal challenge	( )	( )	( )	( )	( )	( )	( )	( )
I would feel bad if I did not	( )	( )	( )	( )	( )	( )	( )	( )
I have chosen for it myself	( )	( )	( )	( )	( )	( )	( )	( )
I think I benefit from it as a patient	( )	( )	( )	( )	( )	( )	( )	( )
I want to show others that I can do this	( )	( )	( )	( )	( )	( )	( )	( )
I find this important	( )	( )	( )	( )	( )	( )	( )	( )
I do not want to disappoint my partner or someone who	( )	( )	( )	( )	( )	( )	( )	( )

is important to me								
I want my doctor to find me an exemplary patient	( )	( )	( )	( )	( )	( )	( )	( )
I think it is important to have insight in my condition myself	( )	( )	( )	( )	( )	( )	( )	( )
I like to have responsibilities within my treatment	( )	( )	( )	( )	( )	( )	( )	( )
I would feel guilty if I did not do it	( )	( )	( )	( )	( )	( )	( )	( )
I really think it's good to do this	( )	( )	( )	( )	( )	( )	( )	( )

The following questions are about what you think of and expect from the Power2DM system.

	Completely disagree	Mostly disagree	Somewhat disagree	Neither agree nor disagree	Somewhat agree	Mostly agree	Completely agree	Not applicable
With the Power2DM system I can really keep an eye on my health	( )	( )	( )	( )	( )	( )	( )	( )
Through the Power2DM system I understand my condition and the treatment better	( )	( )	( )	( )	( )	( )	( )	( )
The Power2DM system has taught me useful things	( )	( )	( )	( )	( )	( )	( )	( )
The Power2DM system clearly provides insight into my current health	( )	( )	( )	( )	( )	( )	( )	( )
With the Power2DM system I identify problems with my condition earlier	( )	( )	( )	( )	( )	( )	( )	( )
With the Power2DM system my health runs less risk	( )	( )	( )	( )	( )	( )	( )	( )

Please indicate to what extent you agree with the statements.

	**Completely disagree**	**Mostly disagree**	**Somewhat disagree**	**Neither agree nor disagree**	**Somewhat agree**	**Mostly agree**	**Completely agree**	**Not applicable**
Working with the Power2DM system takes little effort	( )	( )	( )	( )	( )	( )	( )	( )
The Power2DM system is easy in daily use	( )	( )	( )	( )	( )	( )	( )	( )
The use of the Power2DM system yields no ambiguities	( )	( )	( )	( )	( )	( )	( )	( )
Learning to deal with the Power2DM system is easy for me	( )	( )	( )	( )	( )	( )	( )	( )
I think my family believes I should continue using the Power2DM system	( )	( )	( )	( )	( )	( )	( )	( )
I think my friends believe I should continue using the Power2DM system	( )	( )	( )	( )	( )	( )	( )	( )

I think my health care providers believe I should continue using the Power2DM system	( )	( )	( )	( )	( )	( )	( )	( )
I think my fellow patients believe I should continue using the Power2DM system	( )	( )	( )	( )	( )	( )	( )	( )

Please indicate to what extent you agree with the statements.

	**Completely disagree**	**Mostly disagree**	**Somewhat disagree**	**Neither agree nor disagree**	**Somewhat agree**	**Mostly agree**	**Completely agree**	**Not applicable**
I find it interesting to work with the Power2DM system	( )	( )	( )	( )	( )	( )	( )	( )
I experience working with the Power2DM system as something unpleasant	( )	( )	( )	( )	( )	( )	( )	( )
I like working with the Power2DM system	( )	( )	( )	( )	( )	( )	( )	( )
Working with the Power2DM system gives me a restless feeling	( )	( )	( )	( )	( )	( )	( )	( )
I am confident that the Power2DM system is working well	( )	( )	( )	( )	( )	( )	( )	( )
I have confidence in the information provided by the Power2DM system	( )	( )	( )	( )	( )	( )	( )	( )
I am confident that my own measurements or	( )	( )	( )	( )	( )	( )	( )	( )

questions answered by me provide sufficient insight into my health situation								
I think that using the Power2DM system puts my privacy at risk	( )	( )	( )	( )	( )	( )	( )	( )

Please indicate to what extent you agree with the statements.

	**Completely disagree**	**Mostly disagree**	**Somewhat disagree**	**Neither agree nor disagree**	**Somewhat agree**	**Mostly agree**	**Completely agree**	**Not applicable**
With the help of the Power2DM system, I play a greater role within my own medical care	( )	( )	( )	( )	( )	( )	( )	( )
The Power2DM system contains a lot of interesting information	( )	( )	( )	( )	( )	( )	( )	( )
I find it an advantage that changes in my condition can be quickly noticed by using the POWER2DM system	( )	( )	( )	( )	( )	( )	( )	( )
I find it a disadvantage that I am more preoccupied about my condition by using the POWER2DM system	( )	( )	( )	( )	( )	( )	( )	( )
I find it an advantage that I can adjust the action plan by myself	( )	( )	( )	( )	( )	( )	( )	( )

I find it an advantage that my healthcare provider has direct access to my measured values or manually entered data	( )	( )	( )	( )	( )	( )	( )	( )

Please indicate to what extent you agree with the statements.

	**Completely disagree**	**Mostly disagree**	**Somewhat disagree**	**Not disagree/ not agree**	**Somewhat agree**	**Mostly agree**	**Completely agree**	**Not applicable**
I find it a disadvantage of the Power2DM system that it takes a lot of my time	( )	( )	( )	( )	( )	( )	( )	( )
A disadvantage of Power2DM is that I have less time for other activities	( )	( )	( )	( )	( )	( )	( )	( )
An advantage of using the Power2DM system is that I have more time for other activities.	( )	( )	( )	( )	( )	( )	( )	( )

The following questions are about your computer skills and whether you have everything at home that is needed to work with the Power2DM system.

Please indicate to what extent you agree with the statements.

	**Completely disagree**	**Mostly disagree**	**Somewhat disagree**	**Neither agree nor disagree**	**Somewhat agree**	**Mostly agree**	**Completely agree**	**Not applicable**
I use my computer daily	( )	( )	( )	( )	( )	( )	( )	( )
I find it a disadvantage that I have to use a mobile phone for the Power2DM system	( )	( )	( )	( )	( )	( )	( )	( )
I use the Internet daily	( )	( )	( )	( )	( )	( )	( )	( )
I have sufficient Internet skills to use the Power2DM system	( )	( )	( )	( )	( )	( )	( )	( )
My computer is excellent for working with the Power2DM system	( )	( )	( )	( )	( )	( )	( )	( )
My Internet connection works well	( )	( )	( )	( )	( )	( )	( )	( )

Where possible, my family helps me with the use of the Power2DM system	( )	( )	( )	( )	( )	( )	( )	( )
Where possible, my friends help with the use of the Power2DM system	( )	( )	( )	( )	( )	( )	( )	( )
I can work well with the Power2DM system without the help of others	( )	( )	( )	( )	( )	( )	( )	( )
I can work well with the Power2DM system without the help of the Power2DM-team	( )	( )	( )	( )	( )	( )	( )	( )
I can work well with the Power2DM system as long as someone is available to help me	( )	( )	( )	( )	( )	( )	( )	( )
I can work well with the Power2DM system as long as nothing abnormal happens	( )	( )	( )	( )	( )	( )	( )	( )

The final questions are about how you intend to use the Power2DM system in the coming period.

Please indicate to what extent you agree with the statements.

	**Completely disagree**	**Mostly disagree**	**Somewhat disagree**	**Neither agree nor disagree**	**Somewhat agree**	**Mostly agree**	**Completely agree**	**Not applicable**
In the coming period I will again fill in the measurements in the Power2DM system at the indicated moments	( )	( )	( )	( )	( )	( )	( )	( )
In the coming period I will look at the action plan in the Power2DM system	( )	( )	( )	( )	( )	( )	( )	( )
I will look closely at the graphs on the measurement page of Power2DM	( )	( )	( )	( )	( )	( )	( )	( )
I will certainly follow the advice given by the action plan	( )	( )	( )	( )	( )	( )	( )	( )
I will consult the information modules if I have medical questions	( )	( )	( )	( )	( )	( )	( )	( )

ESM Tables

ESM Table 1a. Lipid values - overall population.

	Baseline (week 0)		Week 11		Week 22		End (week 37)		P-value
	Power2DM	Usual care	Power2DM	Usual care	Power2DM	Usual care	Power2DM	Usual care	
**Total cholesterol, mean (SD), mmol/l**	4.45 (0.92)	4.65 (0.94)	4.42 (0.97)	4.63 (0.93)	4.42 (0.89)	4.71 (1.04)	4.34 (0.81)	4.55 (0.96)	0.52
**LDL cholesterol, mean (SD), mmol/l**	2.41 (0.77)	2.59 (0.85)	2.33 (0.78)	2.59 (0.86)	2.34 (0.75)	2.55 (0.90)	2.29 (0.67)	2.51 (0.77)	0.74
**HDL cholesterol, mean (SD), mmol/l**	1.41 (0.44)	1.45 (0.47)	1.40 (0.46)	1.46 (0.51)	1.49 (0.48)	1.51 (0.51)	1.46 (0.55)	1.55 (0.63)	0.91
**Triglycerides, mean (SD), mmol/l**	1.50 (2.13)	1.39 (1.13)	1.57 (1.83)	1.32 (0.89)	1.59 (1.99)	1.40 (0.95)	1.54 (1.54)	1.40 (0.87)	0.53

POWER2DM: n=111, usual care: n=115. P-value represents the between-group difference in effect over the study period between the POWER2DM and usual care group.
HDL: high density lipoprotein; l: liter; LDL: low density lipoprotein; mmol: millimol; SD: standard deviation.

ESM Table 1b. Lipid values – patients with type 1 diabetes.

	Baseline (week 0)		Week 11		Week 22		End (week 37)		P-value
	Power2DM	Usual care	Power2DM	Usual care	Power2DM	Usual care	Power2DM	Usual care	
**Total cholesterol, mean (SD), mmol/l**	4.42 (0.81)	4.89 (0.73)	4.60 (0.89)	4.81 (0.71)	4.55 (0.86)	4.99 (0.75)	4.50 (0.80)	4.91 (0.85)	0.46
**LDL cholesterol, mean (SD), mmol/l**	2.37 (0.62)	2.74 (0.71)	2.40 (0.77)	2.69 (0.69)	2.33 (0.73)	2.64 (0.70)	2.36 (0.70)	2.69 (0.69)	0.37
**HDL cholesterol, mean (SD), mmol/l**	1.66 (0.44)	1.73 (0.45)	1.67 (0.46)	1.68 (0.45)	1.76 (0.42)	1.82 (0.50)	1.74 (0.61)	1.82 (0.55)	0.65
**Triglycerides, mean (SD), mmol/l**	0.83 (0.36)	0.94 (0.60)	1.03 (0.62)	0.97 (0.52)	1.09 (0.62)	1.10 (0.71)	1.05 (0.44)	1.06 (0.62)	0.15

POWER2DM: n=54, usual care: n=54. P-value represents the between-group difference in effect over the study period between the POWER2DM and usual care group.
HDL: high density lipoprotein; l: liter; LDL: low density lipoprotein; mmol: millimol; SD: standard deviation.

ESM Table 1c. Lipid values - patients with type 2 diabetes.

	Baseline (week 0)		Week 11		Week 22		End (week 37)		P-value
	Power2DM	Usual care	Power2DM	Usual care	Power2DM	Usual care	Power2DM	Usual care	
**Total cholesterol, mean (SD), mmol/l**	4.47 (1.03)	4.45 (1.06)	4.27 (1.02)	4.45 (1.06)	4.27 (0.90)	4.47 (1.20)	4.16 (0.79)	4.24 (0.95)	0.18
**LDL cholesterol, mean (SD), mmol/l**	2.45 (0.89)	2.46 (0.94)	2.26 (0.79)	2.49 (0.98)	2.34 (0.78)	2.48 (0.27)	2.23 (0.65)	2.34 (0.81)	0.29
**HDL cholesterol, mean (SD), mmol/l**	1.17 (0.28)	1.20 (0.31)	1.16 (0.29)	1.26 (0.49)	1.25 (0.40)	1.23 (0.33)	1.20 (0.31)	1.31 (0.60)	0.63
**Triglycerides, mean (SD), mmol/l**	2.13 (2.82)	1.78 (1.32)	2.08 (2.39)	1.63 (1.03)	2.07 (2.64)	1.67 (1.06)	2.00 (2.00)	1.69 (0.96)	0.91

POWER2DM: n=57, usual care: n=61. P-value represents the between-group difference in effect over the study period between the POWER2DM and usual care group.
HDL: high density lipoprotein; l: liter; LDL: low density lipoprotein; mmol: millimol; SD: standard deviation.

ESM Figures

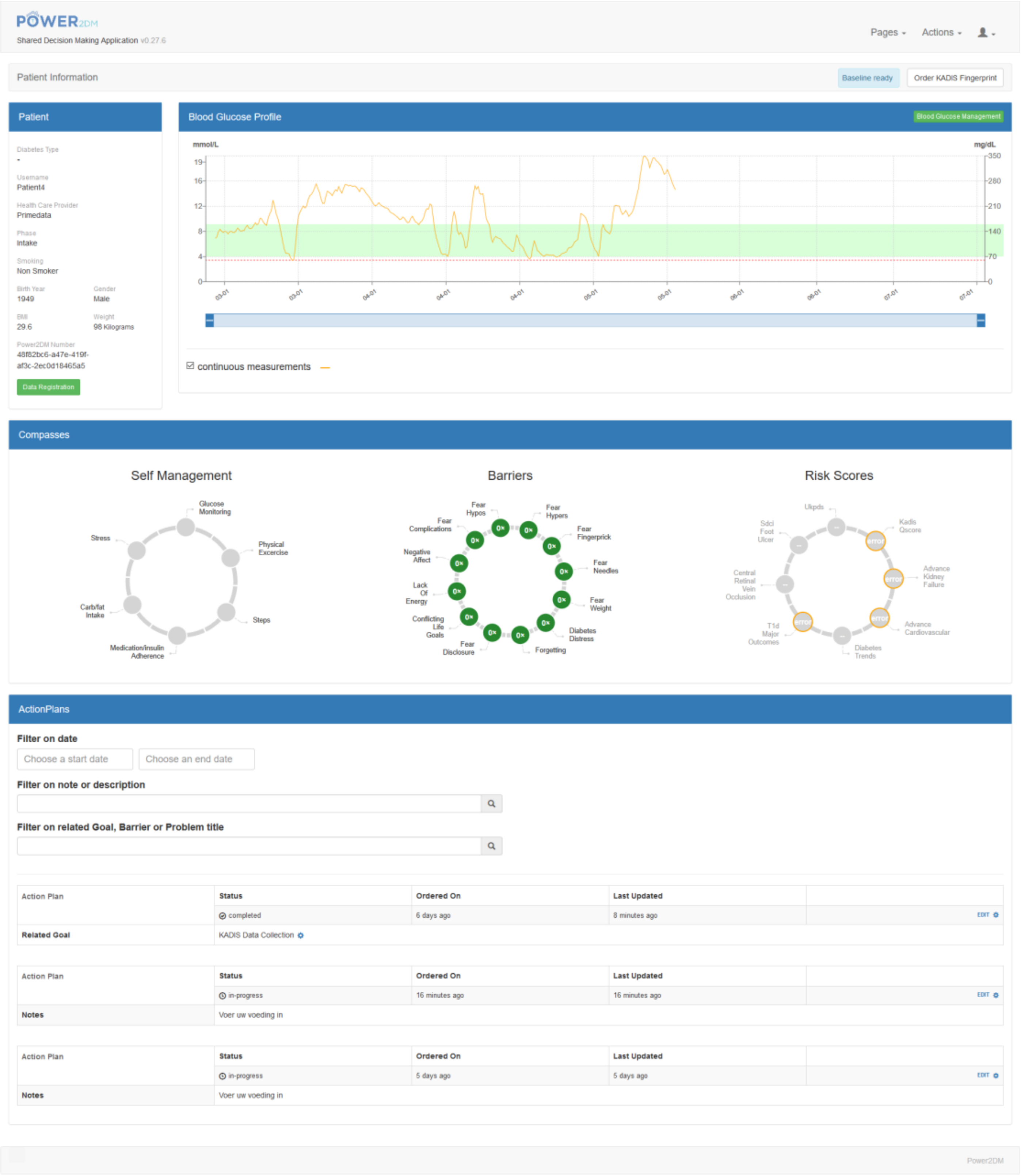

ESM Figure 1: Example of opening screen Shared Decision Making Dashboard (SDMD) application.

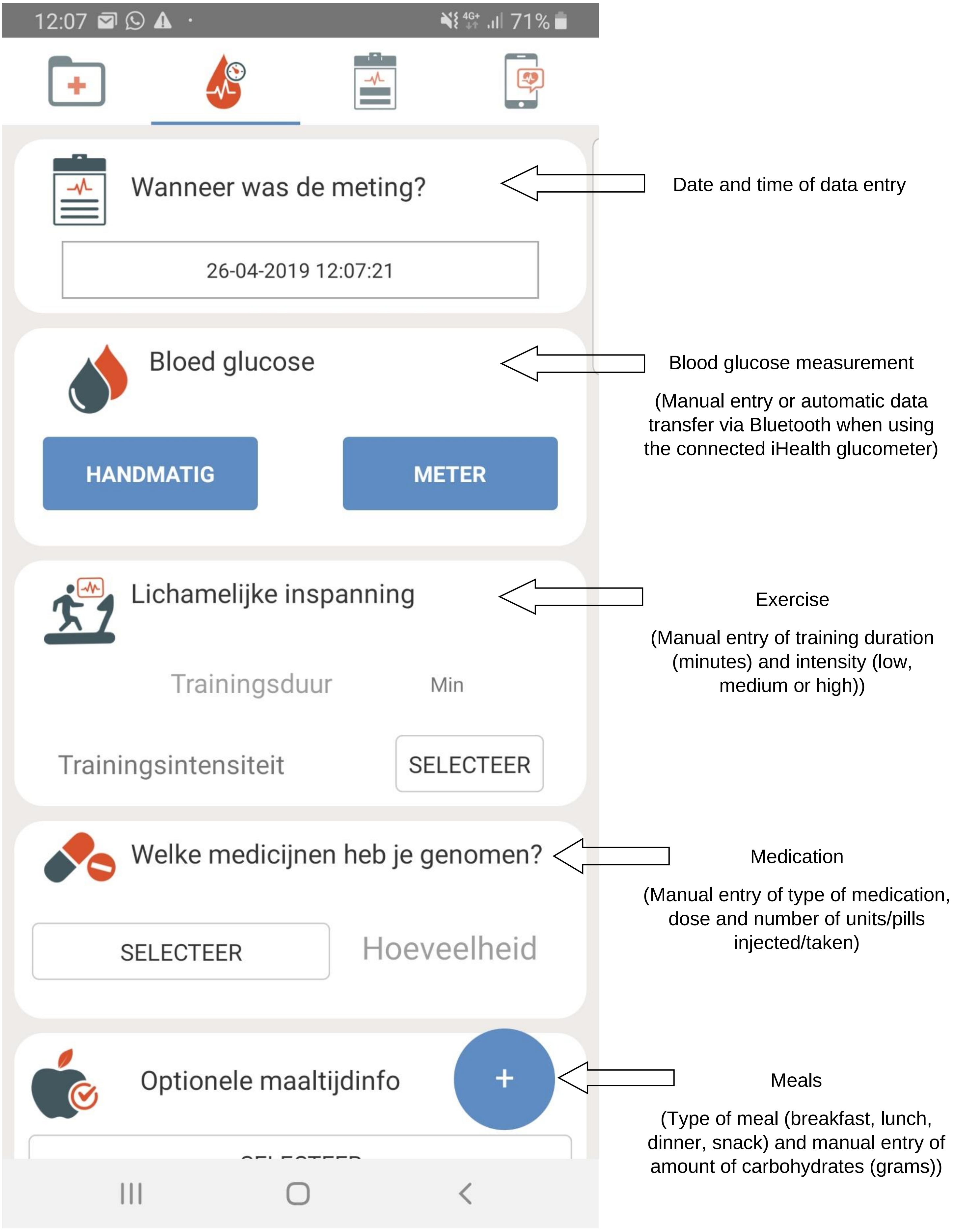

ESM Figure 2: Example of opening screen Self-Management Support System (SMSS) mobile application.

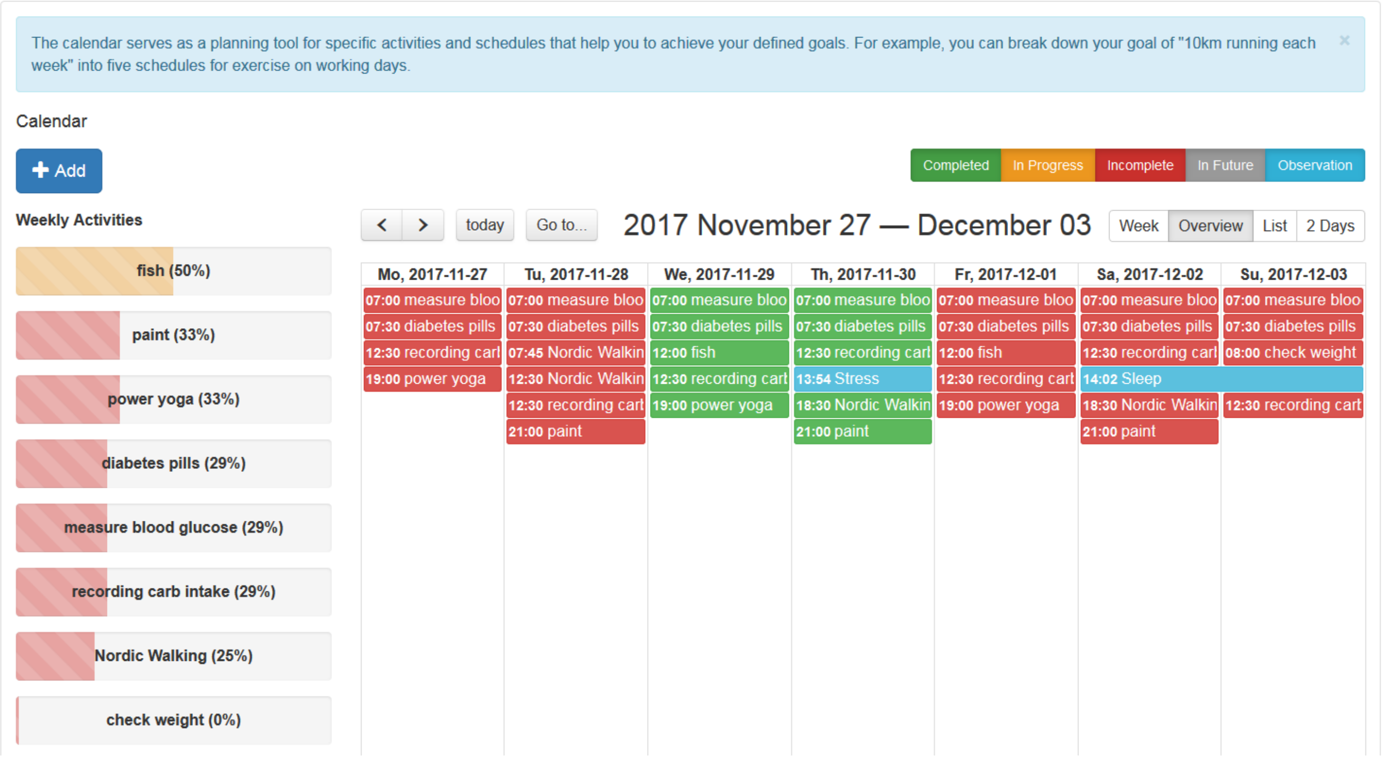

ESM Figure 3: Example of test participant calender in Self-Management Support System (SMSS) application.

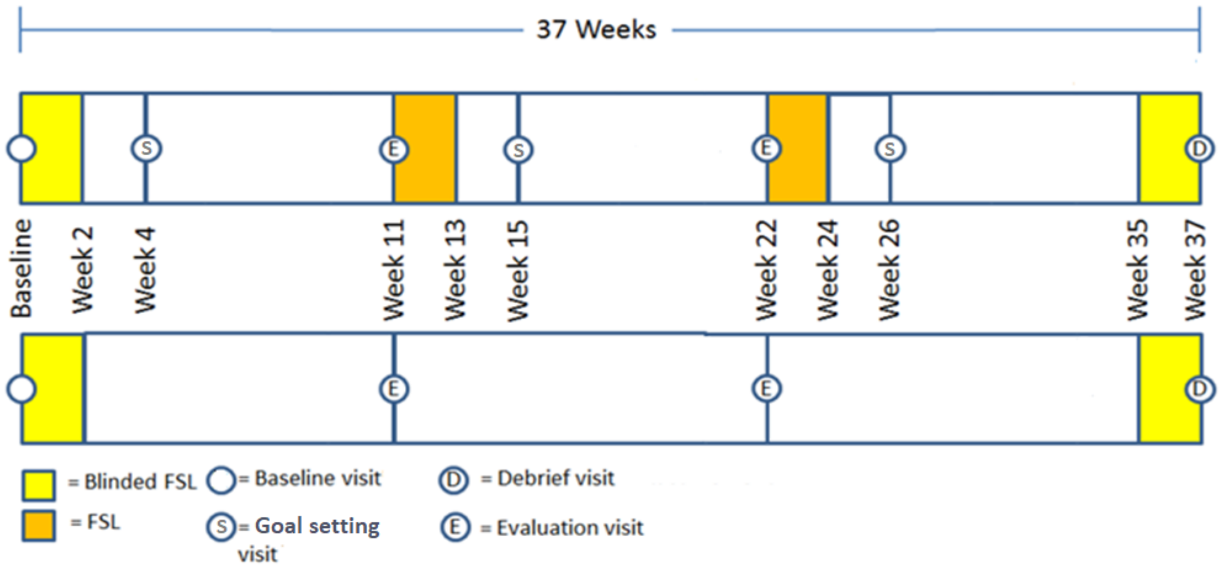

ESM Figure 4: POWER2DM study visit flow chart.
Upper bar: POWER2DM group. Lower bar: usual care group. Blinded FSL: FreeStyle Libre Pro (blinded continuous glucose monitoring device); FSL: FreeStyle Libre (non-blinded intermittently scanned continuous glucose monitoring device).

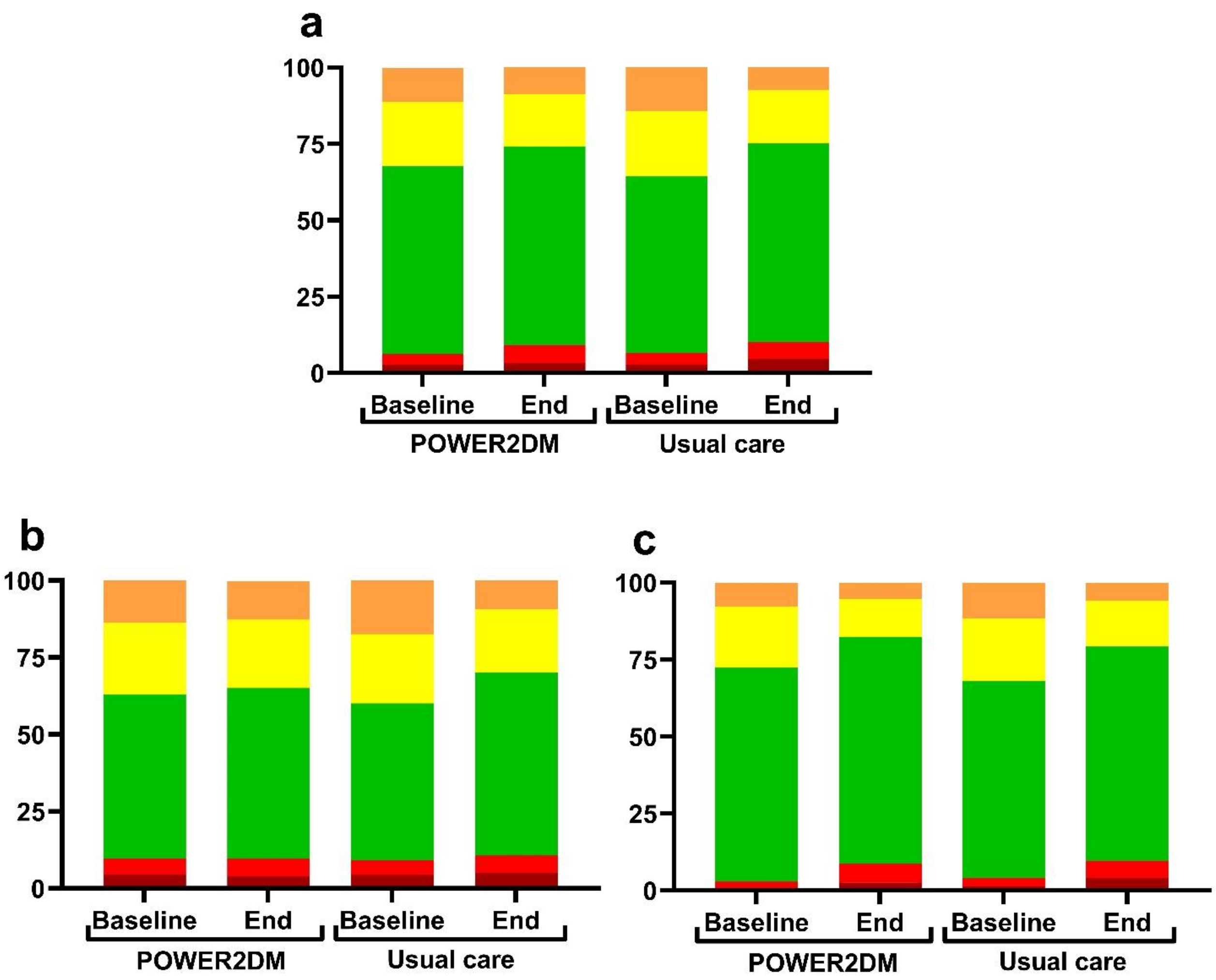

ESM Figure 5: Outcomes of blinded continuous glucose monitoring. a. Outcomes of continuous glucose monitoring measured by a blinded glucose monitoring device (FreeStyle Libre Pro) in POWER2DM and usual care group. b. Outcomes of blinded continuous glucose monitoring in patients with type 1 diabetes in the POWER2DM and usual care group. c. Outcomes of blinded continuous glucose monitoring in patients with type 2 diabetes in the POWER2DM and usual care group. Orange: % of time >13.9 mmol/L. Yellow: % of time 10.1-13.9 mmol/L. Green: % of time 3.9-10.0 mmol/L. Bright red: % of time 3.0-3.8 mmol/L. Dark red: % of time < 3.0 mmol/L.

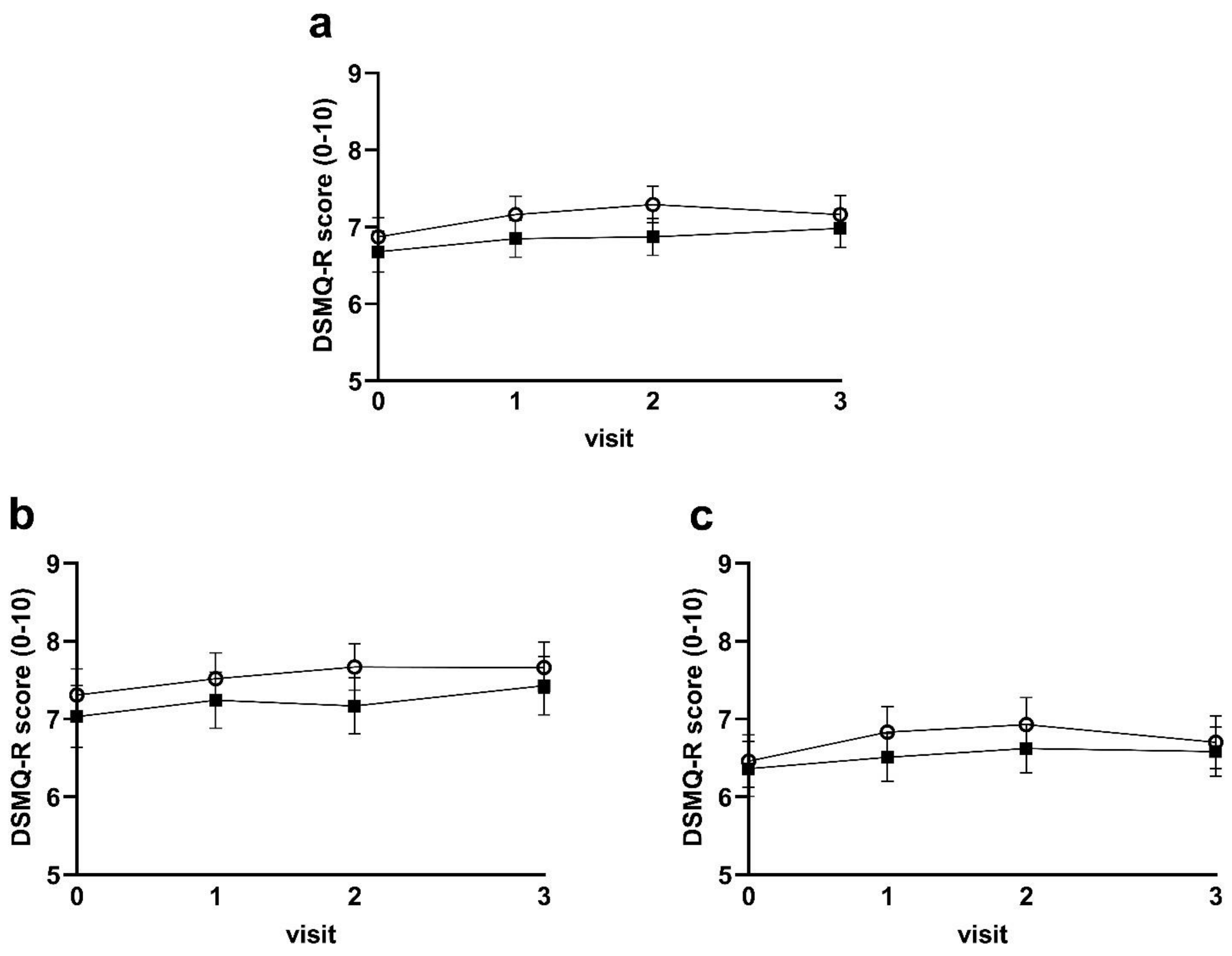

ESM Figure 6: Diabetes self-management. a) DSMQ-R score (diabetes self-management) over the course of the study (POWER2DM: n=111, usual care: n=115). b) DSMQ-R score over the course of the study in patients with type 1 diabetes (POWER2DM: n=54, usual care: n=54). c) DSMQ-R score over the course of the study in patients with type 2 diabetes (POWER2DM: n=57, usual care: n=61). Data are mean, 95% CI. White circles represent the POWER2DM group. Black squares represent the usual care group.

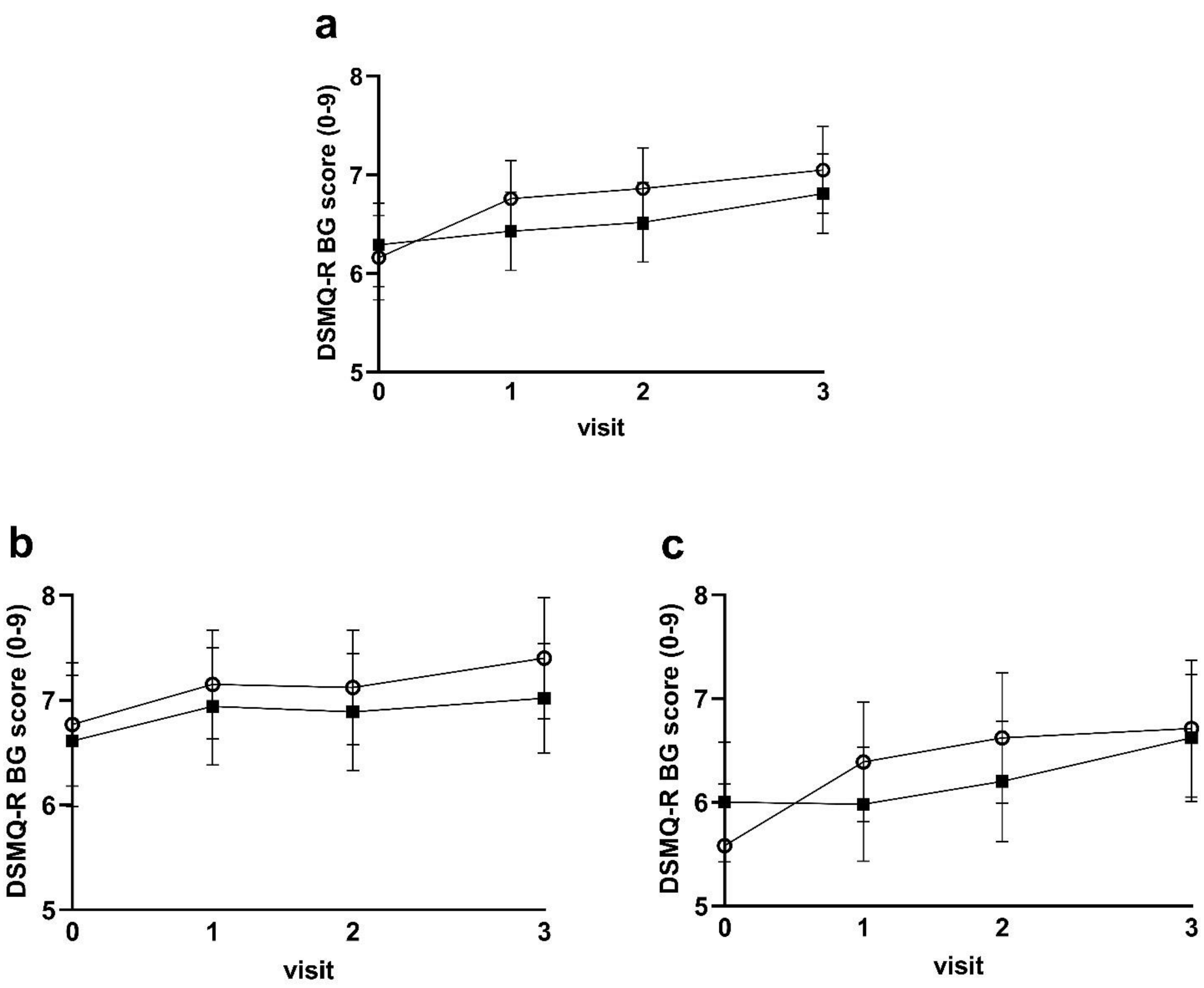

ESM Figure 7: Diabetes self-management of blood glucose. a) DSMQ-R BG score (diabetes self-management of blood glucose) over the course of the study (POWER2DM: n=111, usual care: n=115). b) DSMQ-R BG score over the course of the study in patients with type 1 diabetes (POWER2DM: n=54, usual care: n=54). c) DSMQ-R BG score over the course of the study in patients with type 2 diabetes (POWER2DM: n=57, usual care: n=61). Data are mean, 95% CI. White circles represent the POWER2DM group. Black squares represent the usual care group.

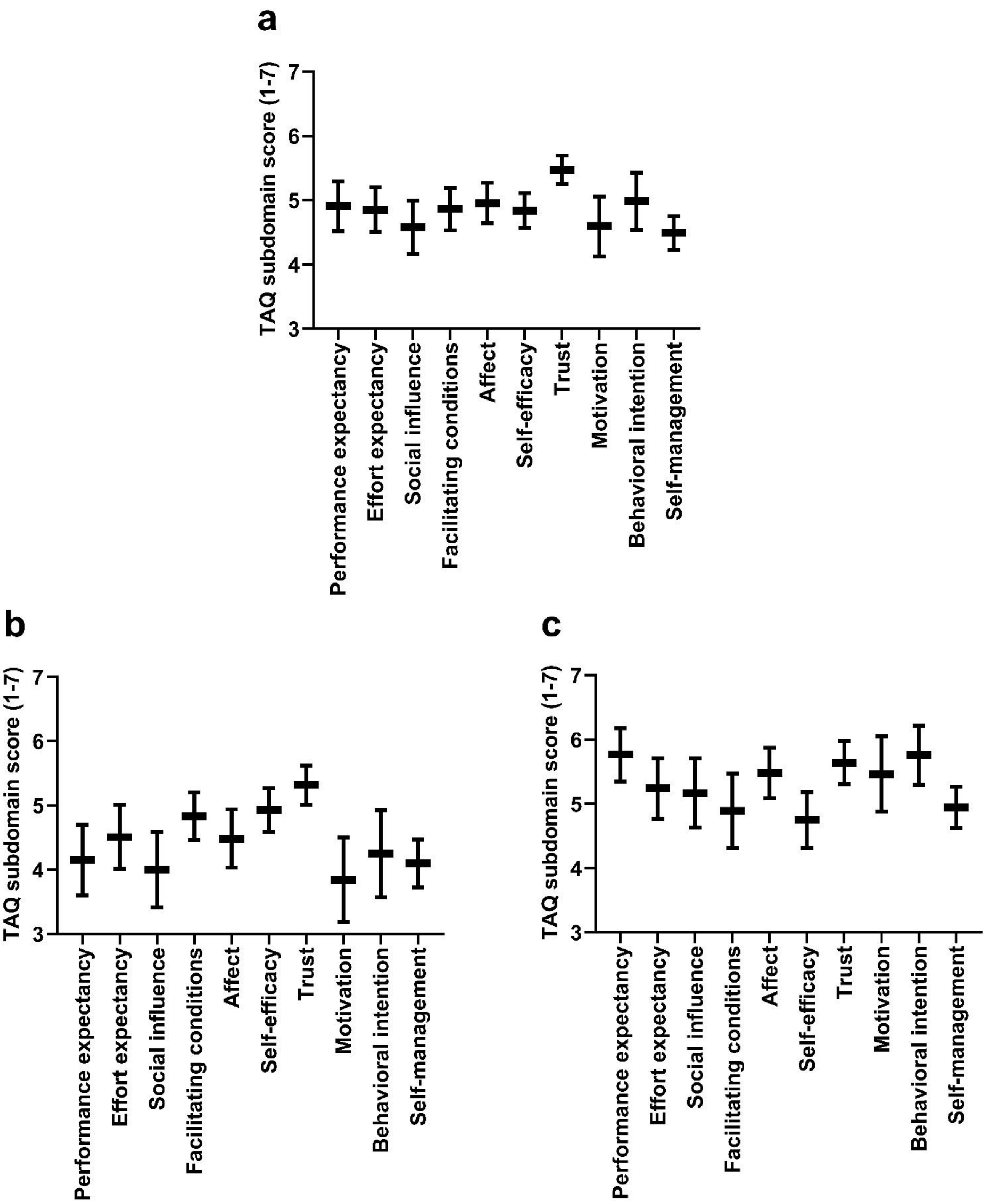

ESM Figure 8: Technology Acceptance.

a) Technology Acceptance Questionnaire (TAQ) subdomain scores of patients in the POWER2DM group at the end of the study (n=84). b) Technology Acceptance Questionnaire (TAQ) subdomain scores of patients with type 1 diabetes in the POWER2DM group at the end of the study (n=45). c) Technology Acceptance Questionnaire (TAQ) subdomain scores of patients with type 2 diabetes in the POWER2DM group at the end of the study (n=39). Data are mean, 95% CI. Performance expectancy: the degree to which patients believe that using the system will help them attain gains or make losses with the performance of their health management. Effort expectancy: the degree of ease associated with the use of the system. Social influence: the degree to which patients perceive that important others believe they should use the system. Facilitating conditions: the

degree to which patients believe that there are objective factors available in their environment to support their use of the system. Affect: patients' overall affective reaction towards the system. Self-efficacy: the degree to which patients judge themselves capable of using the system to manage their health. Trust: the degree to which patients believe that using the system will occur in a safe and reliable manner. Behavioral intention: the degree to which an individual intends to use the POWER2DM system for managing their health. Motivation: the degree to which an individual is motivated to continue the POWER2DM system for managing their health. Self-management: patients' opinion on conducting self-management through the system

References

[1] Welch GW, Jacobson AM, Polonsky WH (1997) The Problem Areas in Diabetes Scale. An evaluation of its clinical utility. Diabetes care 20(5): 760-766. 10.2337/diacare.20.5.760

[2] Snoek FJ, Mollema ED, Heine RJ, Bouter LM, van der Ploeg HM (1997) Development and validation of the diabetes fear of injecting and self-testing questionnaire (D-FISQ): first findings. Diabetic medicine : a journal of the British Diabetic Association 14(10): 871-876. 10.1002/(sici)1096-9136(199710)14:10<871::Aid-dia457>3.0.Co;2-y

[3] Clarke WL, Cox DJ, Gonder-Frederick LA, Julian D, Schlundt D, Polonsky W (1995) Reduced Awareness of Hypoglycemia in Adults With IDDM: A prospective study of hypoglycemic frequency and associated symptoms. Diabetes care 18(4): 517-522. 10.2337/diacare.18.4.517

[4] Gonder-Frederick LA, Schmidt KM, Vajda KA, et al. (2011) Psychometric Properties of the Hypoglycemia Fear Survey-II for Adults With Type 1 Diabetes. Diabetes care 34(4): 801-806. 10.2337/dc10-1343

[5] Taylor E, Crawford J, Gold A (2005) Design and development of a scale measuring fear of complications in type 1 diabetes. Diabetes/metabolism research and reviews 21: 264-270. 10.1002/dmrr.524

[6] Wang W, van Lint CL, Brinkman W-P, et al. (2017) Renal transplant patient acceptance of a self-management support system. BMC Medical Informatics and Decision Making 17(1): 58. 10.1186/s12911-017-0456-y

[7] Battelino T, Danne T, Bergenstal RM, et al. (2019) Clinical Targets for Continuous Glucose Monitoring Data Interpretation: Recommendations From the International Consensus on Time in Range. Diabetes care: dci190028. 10.2337/dci19-0028

[8] White IR, Royston P, Wood AM (2011) Multiple imputation using chained equations: Issues and guidance for practice. Stat Med 30(4): 377-399. 10.1002/sim.4067

[9] Bell ML, Fairclough DL (2014) Practical and statistical issues in missing data for longitudinal patient-reported outcomes. Stat Methods Med Res 23(5): 440-459. 10.1177/0962280213476378
